# Predicting whole genome protein interaction networks from primary sequence data in model and non-model organisms using ENTS

**DOI:** 10.1186/1471-2164-14-608

**Published:** 2013-09-10

**Authors:** Eli Rodgers-Melnick, Mark Culp, Stephen P DiFazio

**Affiliations:** 1 Department of Biology, West Virginia University, Morgantown, West Virginia, 26506, USA; 2 Department of Statistics, West Virginia University, Morgantown, West Virginia, 26506, USA

## Abstract

**Background:**

The large-scale identification of physical protein-protein interactions (PPIs) is an important step toward understanding how biological networks evolve and generate emergent phenotypes. However, experimental identification of PPIs is a laborious and error-prone process, and current methods of PPI prediction tend to be highly conservative or require large amounts of functional data that may not be available for newly-sequenced organisms.

**Results:**

In this study we demonstrate a random-forest based technique, ENTS, for the computational prediction of protein-protein interactions based only on primary sequence data. Our approach is able to efficiently predict interactions on a whole-genome scale for any eukaryotic organism, using pairwise combinations of conserved domains and predicted subcellular localization of proteins as input features. We present the first predicted interactome for the forest tree *Populus trichocarpa* in addition to the predicted interactomes for *Saccharomyces cerevisiae*, *Homo sapiens*, *Mus musculus*, and *Arabidopsis thaliana*. Comparing our approach to other PPI predictors, we find that ENTS performs comparably to or better than a number of existing approaches, including several that utilize a variety of functional information for their predictions. We also find that the predicted interactions are biologically meaningful, as indicated by similarity in functional annotations and enrichment of co-expressed genes in public microarray datasets. Furthermore, we demonstrate some of the biological insights that can be gained from these predicted interaction networks. We show that the predicted interactions yield informative groupings of *P. trichocarpa* metabolic pathways, literature-supported associations among human disease states, and theory-supported insight into the evolutionary dynamics of duplicated genes in paleopolyploid plants.

**Conclusion:**

We conclude that the ENTS classifier will be a valuable tool for the *de novo* annotation of genome sequences, providing initial clues about regulatory and metabolic network topology, and revealing relationships that are not immediately obvious from traditional homology-based annotations.

## Background

Proteins do not exist within a vacuum. Much of the startling diversity of living organisms emerges only with the aggregate combinatorial complexity of protein-protein interactions (PPIs) [1]. As such, the discovery of physical interactions between proteins is often an essential step in the characterization of protein functions, providing insights into diverse cellular processes such as the fluxes of metabolic pathways, the logic of transcriptional activation, and the kinetics of signal transduction. This in turn forms the basis of understanding biological functions at the organismal scale, including mechanisms of environmental responses and the etiology of disease states.

Despite the biological importance of PPIs and the availability of high-throughput screening methods in recent years, experimentally-verified PPI networks remain sparsely populated, especially with respect to the amount of sequence data currently available. High throughput approaches such as automated yeast two-hybrid screens and tandem affinity purification/mass spectrometry have detected thousands of binary PPIs in animal and fungal model organisms such as *Homo sapiens*[2], *Saccharomyces cerevisiae*[3], and *Drosophila melanogaster*[4], yet the current size of the interactome belonging to the experimental workhorse of the plant kingdom, *Arabidopsis thaliana*, only constitutes approximately 3% of its expected size [5]. Moreover, the lack of significant numbers of PPIs for non-model species hinders the development of evolutionary studies concerning rewiring within the interactome [6].

The demand for additional PPIs has led to the development of several methods for computational PPI prediction over the past decade. Several groups have attempted to expand the *A. thaliana* interactome using statistical learning methodology and/or transfer of interaction annotation based on homology (interologs) [7-10]. Similar methods have been used to expand the number of network connections in the *S. cerevisiae* and *H. sapiens* proteomes. Though each of these methods does have the potential to provide useful network information, each approach carries distinct disadvantages limiting its use on non-model species. The interolog-based approaches are limited to discovering PPIs for only the most conserved proteins, as reflected by their relatively low discovery rates in *A. thaliana*[9]. Other methods rely on an ensemble of functional data, such as genome-wide measures of co-expression and co-localization, which is often not available for non-model organisms.

Although many homologous proteins may evolve to become highly dissimilar at the primary sequence level, they often retain conserved structural and/or functional units known as domains. These domains may directly mediate interactions between proteins, as demonstrated by databases of domain-domain interactions such as DOMINE [11]. However, even in the absence of direct interaction, certain pairwise combinations of domains suggest a high probability of interaction. Thus, domain-based approaches of PPI prediction have the potential to provide the advantages of the interolog-based approaches while maintaining utility for less-conserved proteins, especially if the approach also includes features more sensitive to fine-scale differences in amino acid content. There have been several recent attempts to infer PPIs based on pairwise domain information. For example, Singhal *et al.* used a genetic algorithm to discover domain-domain interactions that could be used as predictors of PPIs [12]. Chen and Liu used a domain-driven random forest classifier to predict PPIs for *S. cerevisiae*[13]. However, feature representation for the algorithm required vectors with several thousand entries, making its use computationally expensive for full-genome prediction. Although these approaches are initially promising, there is a lack of publicly-available software that would enable domain-based PPI prediction on a genome-wide scale for non-model organisms lacking large experimental data sets.

Here we present “Elucidating Network Topology with Sequence” (ENTS), a binary PPI classifier that uses a random forest framework. ENTS is capable of efficiently and exhaustively evaluating all potential protein-protein pairs in a large eukaryotic genome using parallelization. We show that the method provides comparable or better predictions on recently experimentally-determined PPIs than several existing methods and that such predictions are biologically plausible using the predicted interactomes of *A. thaliana*, *P. trichocarpa*, *M. musculus*, *H. sapiens*, and *S. cerevisiae*. Scripts, instructions for use, and predicted PPIs for several organisms are available at http://ENTS.as.wvu.edu.

## Results

### ENTS performance relative to experimental predictions

**Figure 1:**
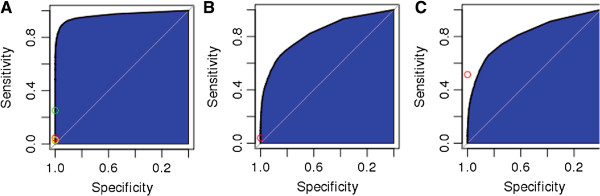
**ENTS performance relative to experimental predictions.** The ROC curves for ENTS testing data on **(A)** *A. thaliana*, **(B)** *H. sapiens*, and **(C)** *S. cerevisiae*. Dots represent measures on the same testing data from alternative predictors, with the De Bodt (red), PAIR (green), AtPID (yellow), and Geisler-Lee (orange) predictors for *A. thaliana*, the PIPS predictor for *H. sapiens*, and the Pitre predictor for *S. cerevisiae*.

We assessed the performance of ENTS by calculating the area underneath the ROC curve (AUC) for testing data consisting of no overlap with the training data at the level of protein interaction and no overlap with any protein pairs used to calculate pairwise domain LOD scores (see methods). AUC scores ranged from 0.811 and 0.827 in the yeast and human-trained classifiers, respectively, to a high of 0.9632 in the *A. thaliana*-trained classifier (Figure 1). The most important features for the classifier included those derived from the analysis of domain pairs and the output from the subcellular localization prediction program multiLoc2 that measures the extent to which the amino acid contents and phylogenetic profiles of the query proteins matched particular cellular compartments (Additional file 1: Figures S6, S7 and S8).

### ENTS performance relative to other classifiers

We obtained whole-genome predictions of PPIs for the organisms on which the classifiers were trained (i.e., *S. cerevisiae*, *H. sapiens* and *A. thaliana*), as well as for species that were not used in training the predictors. We find that the numbers of predicted interactions are highly similar between the training and prediction species, although the training species do have an enrichment of genes at the high confidence levels (Table 1).

**Table 1:** Whole genome prediction counts

**Training**	**Prediction**	**0.55**	**0.65**	**0.75**	**0.85**
*S. cerevisiae*	*S. cerevisiae*	29,616 (4,320)	10,933 (3,314)	2,841 (1,713)	497 (576)
*H. sapiens*	*H. sapiens*	212,365 (12,936)	94,082 (9,906)	29,562 (6,377)	4,180 (2,223)
	*M. musculus*	244,548 (13,615)	98,108 (10,157)	26,860 (5,889)	2,825 (1,496)
*A. thaliana*	*A. thaliana*	346,020 (15,964)	176,600 (13,426)	79,796 (9,504)	19,915 (4,010)
	*P. trichocarpa*	481,253 (19,321)	178,232 (14,536)	42,503 (7,501)	4,085 (1,316)

Protein-protein interactions predicted using ENTS at several confidence cutoffs. The number of genes involved in the predicted interactions is shown in parentheses.

Although most alternative classifiers performed similarly to ENTS on the testing data set (Figure 1), this set included interactions that were used to train the alternatives and could thereby inflate their sensitivities at a given specificity value. Therefore, in order to assess the performance of ENTS on whole genome data relative to several alternative classifiers, we examined the frequency of positive predictions among sets of experimentally-determined PPIs that were not used for training, testing, or calculation of pairwise domain odds in ENTS or used for training in the alternative classifiers. We obtained genome-wide predicted PPI datasets in *S. cerevisiae*[14], *H. sapiens*[15], *M. musculus*[16], and *A. thaliana*[7-10]. The sizes of the predicted datasets varied greatly, so when making comparisons we reduced the sizes of the ENTS predictions to those of the alternative datasets following removal of predictions that corresponded to data used for ENTS training or calculation of pairwise domain odds in order to place bounds on the possible number of positive predictions and thereby provide fair comparisons.

**Figure 2:**
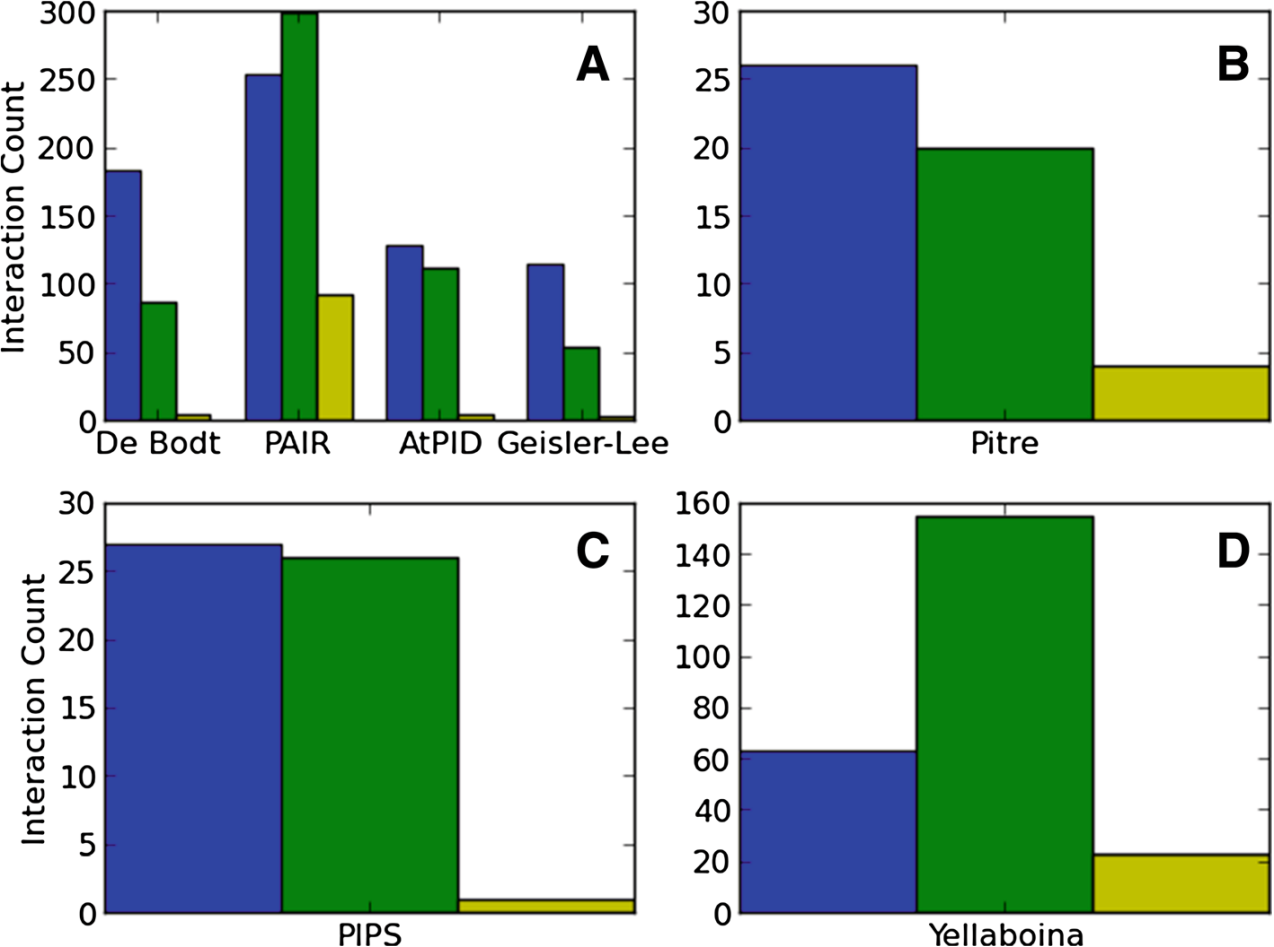
**ENTS performance relative to other classifiers.** The number of predictions supported by experiment on sets of novel experimentally-determined interactions for ENTS and several alternative prediction techniques in **(A)** *A. thaliana*, **(B)** *S. cerevisiae*, **(C)** *H. sapiens*, and **(D)** *M. musculus*. ENTS results are shown in blue; the alternative classifiers’ results are shown in green; and the number of positive predictions shared between each pair of classifiers is shown in yellow.

For *A. thaliana*, we compared predictions to 6,314 novel yeast two-hybrid and literature-curated PPIs from a large-scale study of interactome evolution [5]. We found that ENTS predicted more of these interactions than 3 of the 4 alternative classifiers (Figure 2A). This included more than twice as many predicted interactions as the Geisler-Lee (*n* = 19,779) and De Bodt (*n* = 51,594) sets, each of which used interolog approaches to make their predictions [7,9]. By contrast, ENTS made a similar number of positive predictions to the AtPID classifier (*n* = 24,248) and less positive predictions than the PAIR classifier (*n* = 143,939). Those two approaches used machine learning techniques - naive Bayes and SVM, respectively - to combine interolog data with domain content and functional data such as co-expression, gene ontology similarity, and co-localization [8,17]. Strikingly, each classifier shared relatively few of its positive predictions with the ENTS predictor, with the highest number of interactions shared between the ENTS and PAIR classifiers, at 36.8% of the ENTS predictions. Due to the high number of novel interactions discovered within this single Y2H experiment, we also used this to assess the frequency of experimentally-supported novel interactions among all interactions that were predicted between proteins within this set (Additional file 1: Table S1). We find that the ratio of experimentally-supported interactions to all positive predictions is higher for ENTS than all alternative classifiers except AtPID. However, the actual true and false positive rates within this set are not possible to obtain due to the low sensitivity (16%) of the Y2H assay [5].

We find that the number of novel interactions predicted by ENTS is similar to those predicted by the Pitre *et al.* PIPE2 classifier (*n* = 13,826) in *S. cerevisiae* and the PIPS classifier (*n* = 22,687) in human [14,15] (Figure 2A-C), as found through comparisons to a high-throughput yeast two-hybrid dataset in *S. cerevisiae*[3] (*n* = 1,337) and high-confidence interactions from two large-scale studies of human PPIs (*n* = 2,045) [18,19]. Again, relatively few predictions were shared between each pair of classifiers. The alternative classifiers differed substantially in their prediction methods. The Pitre *et al.* classifier based its predictions on the pairwise-occurrence of short sequence motifs [14], while the PIPS classifier used naive Bayes to combine sequence-derived features such as orthology and pairwise domain content with functional data such as gene co-expression, post-translational modifications, and co-localization [15]. In mouse, we find that ENTS predicted many fewer novel interactions from a high-confidence set of literature-curated interactions (*n* = 1,807) relative to the interolog-based predictor of Yellaboina *et al.* (*n* = 36,608) (Figure 2D). The high number of mouse interologs inferred directly from human interactions is responsible for most of this disparity, as ENTS predicts more of the novel interactions (36 vs. 25) when the human-derived interologs are filtered out of the Yellaboina *et al.* dataset.

Although training was performed with the response defined as the presence of an interaction between two proteins, we also repeated the comparisons after restricting to a protein set that did not occur within the data used for calculation of domain odds or training the classifier. We did this in order to assess the ability of ENTS to predict beyond the scope of proteins for which there is interaction data currently available. For the species in which there were large numbers of these proteins among the novel interactions - *A. thaliana* (*n = 2,239*) and *M. musculus* (*n = 1,005*), we observe a decline in the relative number of novel interactions predicted relative to other classifiers (Additional file 1; Figure S1), although the predicted interactions shared between ENTS and the alternatives remain low. The remaining two species contained relatively few proteins with novel interactions that were never used for calculation of domain odds (*H. sapiens n = 305*; *S. cerevisiae n = 208*). Neither the truncated ENTS set nor the PIPE2 classifier predicted any interactions within this *S. cerevisiae* set. ENTS did predict two of the novel interactions within the *H. sapiens* set, while the PIPS classifier failed to predict any novel interactions within this set.

### Biological plausibility of PPI predictions

**Figure 3:**
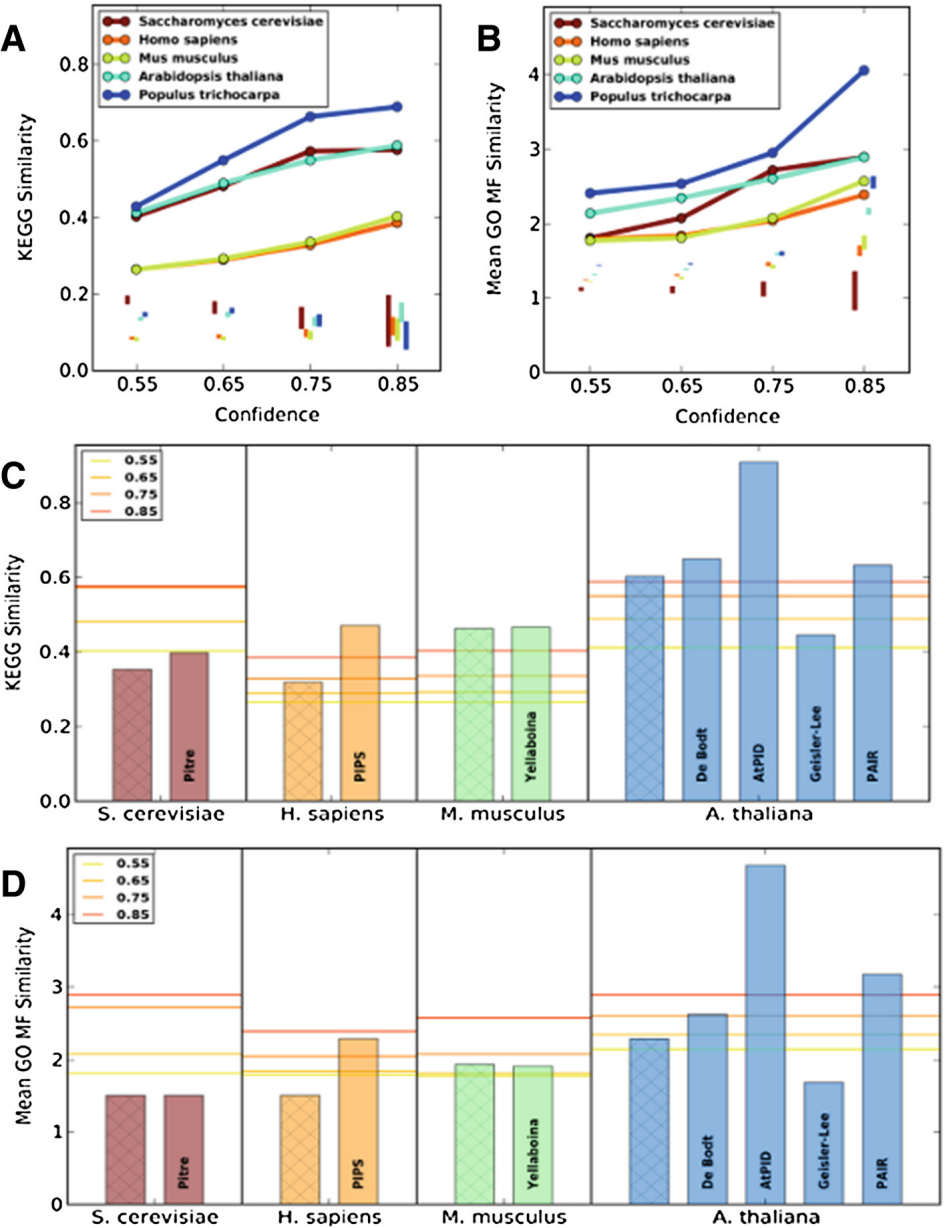
**Biological similarity.** **(A)** The frequency of shared KEGG pathways and **(B)** the mean GO molecular function similarity scores for predicted ENTS interactions. Vertical lines at each confidence level show the total range for randomized networks. **(C)** Shared KEGG pathway frequency and **(D)** mean GO molecular function similarity for ENTS as compared to other predicted networks and the experimentally-verified network. The experimentally-verified networks are shown on the left for each organism, with a hatched bar.

The majority of predicted interactions for each organism are not experimentally verified, so we required indirect means of assessing their plausibility. This led us to assess the similarity of annotations and expression profiles among predicted interactors, excluding self-interactions to avoid upward bias. We find that ENTS-predicted interactors share KEGG pathways significantly more often than expected by chance for all species (Figure 3A). Moreover, KEGG and GO similarity for the organisms with the largest experimentally-determined interactomes - *H. sapiens* and *S. cerevisiae* - matches or exceeds those of the experimentally-verified networks (Figure 3C,D). *M. musculus* KEGG similarity closely matches that observed for *H. sapiens*, while the measures for *P. trichocarpa* actually exceed those of *A. thaliana* at higher confidence levels (Figure 3A). We also find that the mean semantic similarities between predicted interactors for GO biological process (BP), GO cellular component (CC), and GO molecular function (MF) are significantly greater than expected by chance (Figure 3B, Additional file 1: Figure S5). However, even though GO categories were not included as predictors during random forest prediction, they were used during subcellular localization prediction, so their use as a verification criterion is somewhat circular.

**Figure 4:**
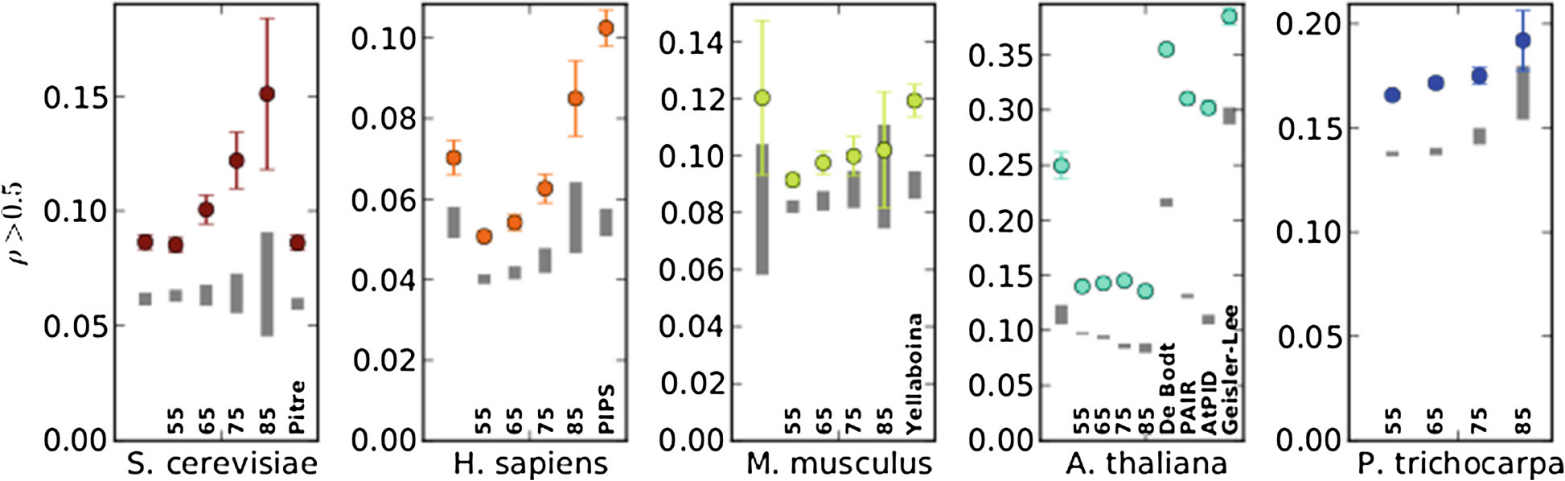
**Significant co-expression.** Dots show the observed frequency of interactors with *ρ* > 0.5. Error bars indicate 95% confidence intervals based on 250 bootstrapped replicates. Grey bars indicate 95% confidence intervals from 250 randomized networks. The leftmost lane for each organism with the exception of *P. trichocarpa* is the data for the experimentally-verified network.

Lastly, we find that the distributions of Pearson gene expression correlations between ENTS-predicted interactors are significantly enriched for co-expressed genes (*ρ* > 0.5) in all organisms (Figure 4). The extent of enrichment varies by organism, with all confidence levels yielding significant enrichment of co-expressed genes in *S. cerevisiae*, *H. sapiens*, and *A. thaliana*. ENTS produces a significant enrichment of co-expressed genes for *M. musculus* at 0.55 and 0.65 confidence levels and for *P. trichocarpa* at all confidence levels except 0.85. Notably, however, the *M. musculus* experimentally-verified network is not significantly enriched for co-expressed genes under the microarray experiment used. Several alternative prediction methods yield networks with much higher co-expression than those predicted by ENTS. However, several of these - PAIR, AtPID, and PIPS - used co-expression as a predictor of protein interaction [8,10,15].

### Metabolic pathway linkages in *P. trichocarpa*

The connectivity of PPI networks permits insight into higher-order structures that largely remain hidden under non network-based analyses. Several studies have demonstrated that biological networks are organized into pathways or modules, each of which contain highly connected groups of genes that may act semi-autonomously with respect to the action of the network as a whole [20]. We used the predicted *P. trichocarpa* PPI network to infer a network of *P. trichocarpa* metabolic pathways, which we then analyzed for higher order structures.

**Figure 5:**
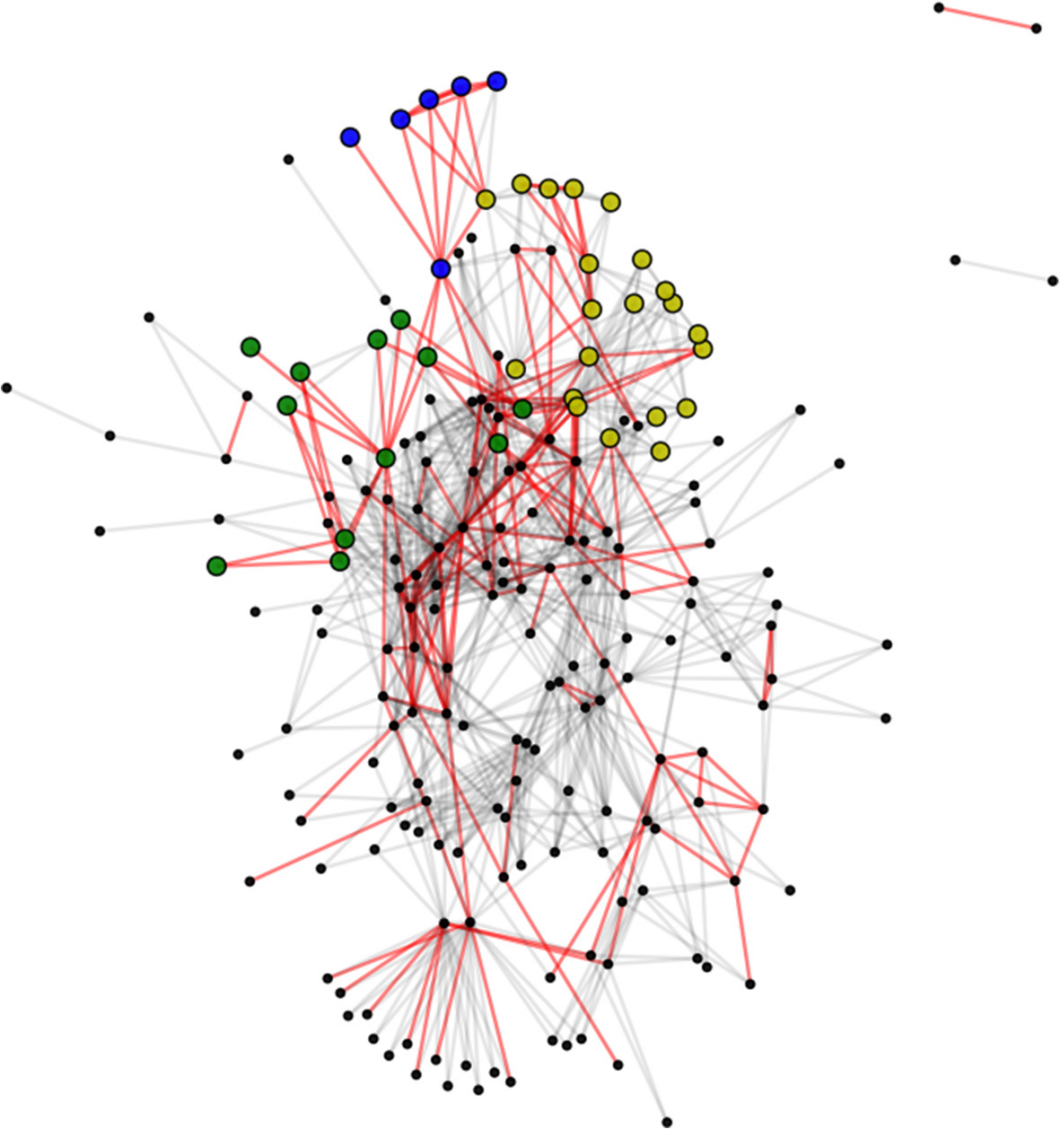
***P. trichocarpa* metabolic linkage network.** Each node represents a pathway in the PoplarCyc metabolic network, with lines connecting those with a significant number interpathway interactions based on ENTs predictions. Red lines indicate a shared compound between pathways. The yellow nodes belong to a cluster significantly enriched for fatty acid biosynthesis, while the green and blue nodes belong to pathways significantly enriched for nucleotide/nucleoside biosynthesis and degradation, respectively.

We produced a network of pathway-pathway associations between poplarCyc v. 3 metabolic pathways [21] by placing edges between pathways that share a significant number of predicted PPIs between the proteins underlying the pathway, excluding predicted self-interactions. Pathway linkages are considered significant if the number of inter-pathway interactions exceeds the number found in 99.9% of randomized networks. Using the ENTS 0.65 *P. trichocarpa* network, we find 913 significant pathway linkages (Figure 5, Additional file 2). All but 2 pairs of pathway linkages are joined within the largest connected component of the linkage graph, and the groups not connected to the primary component include one pair of sulfate metabolic pathways and one pair of heavy metal transporters. Out of the 913 total linkages, we find 173 that share at least 1 compound, significantly more than expected by chance (85.95 ± 7.90, *p* < 0.0001). Seven out of the 10 most highly connected pathways are involved in the biosynthesis of carbohydrates, with the sucrose biosynthesis pathway having the highest degree with 38 pathway linkages. The major entry point of reduced nitrogen, the glutamine biosynthesis pathway, is the fifth most highly connected pathway with 27 linkages. The remaining 2 most highly connected pathways include the flavonoid biosynthesis pathway and glycolysis.

We then used the MCL algorithm to produce clusters of pathways, which we assessed for enrichment of specific pathway classes (Table 2, Additional file 3). We find 9 out of 22 clusters that are significantly enriched for specific classes at a family-wise error rate of 0.05, following a Bonferroni correction. The largest cluster is highly enriched for the biosynthesis of phenylpropanoid derivatives (*p* = 4.105 × 10^−11^), which include a large variety of secondary metabolites important for structure, defense against pathogens, and defense from herbivory. A second cluster is highly enriched for the biosynthesis of fatty acids and lipids (*p* = 2.838 × 10^−10^) (Figure 5). The fatty acid biosynthesis cluster includes not only pathways for the production of phospholipds - primarily phosphatidylcholine - but also biosynthetic pathways for hydrophobic electron carriers such as quinones and quinols. This cluster also contains the rubisco shunt, which acts as a bypass to the Calvin cycle in order to decrease carbon loss during carbohydrate to lipid conversion by approximately 40% [22]. The other pathways in this cluster include the two glycolysis pathways, which act as the other main sources of pyruvate prior to fatty acid synthesis. Two additional clusters are highly enriched for nucleoside/nucleotide biosynthesis (*p* = 1.831 × 10^−13^) and nucleoside/nucleotide degradation (*p* = 2.283 × 10^−21^), respectively (Figure 5). Interestingly, while the degradation cluster only contains purine and pyrimidine degradation pathways and a single pyrimidine salvage pathway, the biosynthesis cluster also contains arginine biosynthesis pathways. This non-intuitive grouping is supported by work in *A. thaliana* that demonstrates the coordination of arginine biosynthesis with the biosynthesis of pyrimidines [23]. Amino acid biosynthesis pathways are divided over several clusters and therefore do not consistently show up as enriched within their clusters. However, the groupings of several amino acid biosynthetic pathways do reflect their biochemical commonalities. The single cluster with significant enrichment of amino acid biosynthesis groups two of the three amino acids derived from 3-phosphoglycerate - serine and cysteine - together with threonine, the only other amino acid besides serine to carry a hydroxyl group. The 3 branched chain amino acid biosynthetic pathways are also grouped together within cluster 9, while the two proline biosynthetic pathways are grouped with glutamine and glutamate biosynthesis in clusters 2 and 4, reflecting their common origins from *α*-ketoglutarate (Table 2, Additional file 3).

**Table 2:** **Significant enrichments in clusters of the *P. trichocarpa * metabolic linkage network**

**Cluster**	**Class**	**p-value**
1 (31)	Phenylpropanoid derivatives biosynthesis (6)	4.105 × 10^−11^
	Secondary metabolites biosynthesis (8)	5.815 × 10^−9^
	Flavonoids biosynthesis (6)	6.486 × 10^−9^
	Carbohydrates biosynthesis (9)	2.185 × 10^−8^
	Sugars biosynthesis (8)	5.65 × 10^−6^
2 (22)	Nitrogen compounds metabolism (3)	1.077 × 10^−5^
	Inorganic nutrients metabolism (3)	1.134×10^−5^
3 (21)	Fatty acids and lipids biosynthesis (8)	2.838 × 10^−10^
	Cofactors, prosthetic groups, electroncarriers biosynthesis (8)	1.741 × 10^−7^
	Phospholipid biosynthesis (6)	2.106 × 10^−7^
	Quinol and quinone biosynthesis (3)	6.301 × 10^−6^
4 (17)	Vitamins biosynthesis (4)	3.871 × 10^−6^
8 (12)	Nucleosides and nucleotides biosynthesis (6)	1.831 × 10^−13^
	Purine nucleotide biosynthesis (3)	7.213 × 10^−6^
11 (6)	Nucleosides and nucleotides degradation (5)	2.283 × 10^−21^
	Purine nucleotides degradation (4)	2.128 × 10^−13^
	Degradation/Utilization/Assimilation (5)	3.977 × 10^−10^
15 (3)	Amino acids biosynthesis (3)	1.642 × 10^−6^
22 (2)	Inorganic nutrients metabolism (2)	2.583 × 10^−7^
	Sulfur compounds metabolism (2)	1.062 × 10^−6^
23 (2)	Transport (2)	1.256 × 10^−5^

Classes of metabolic pathway that were significantly enriched in clusters at a 0.05 family-wide type I error rate. The number of pathways present in each cluster is given in parentheses in the first column, while the number of the given class of pathway within each cluster is given by parentheses in the second column.

### Predictions of human disease associations

The analysis of PPI networks has great potential for aiding our understanding of heritable disease, as the manifestation of a given pathology may result from the perturbation of entire network modules rather than the abrogation of a single gene [24]. In particular, the physical associations between disease-related genes within a protein interaction network may signify a functional relationship between the corresponding disease states, including co-morbidity or alternative routes to a disease due to disruption of a shared pathway.

**Figure 6:**
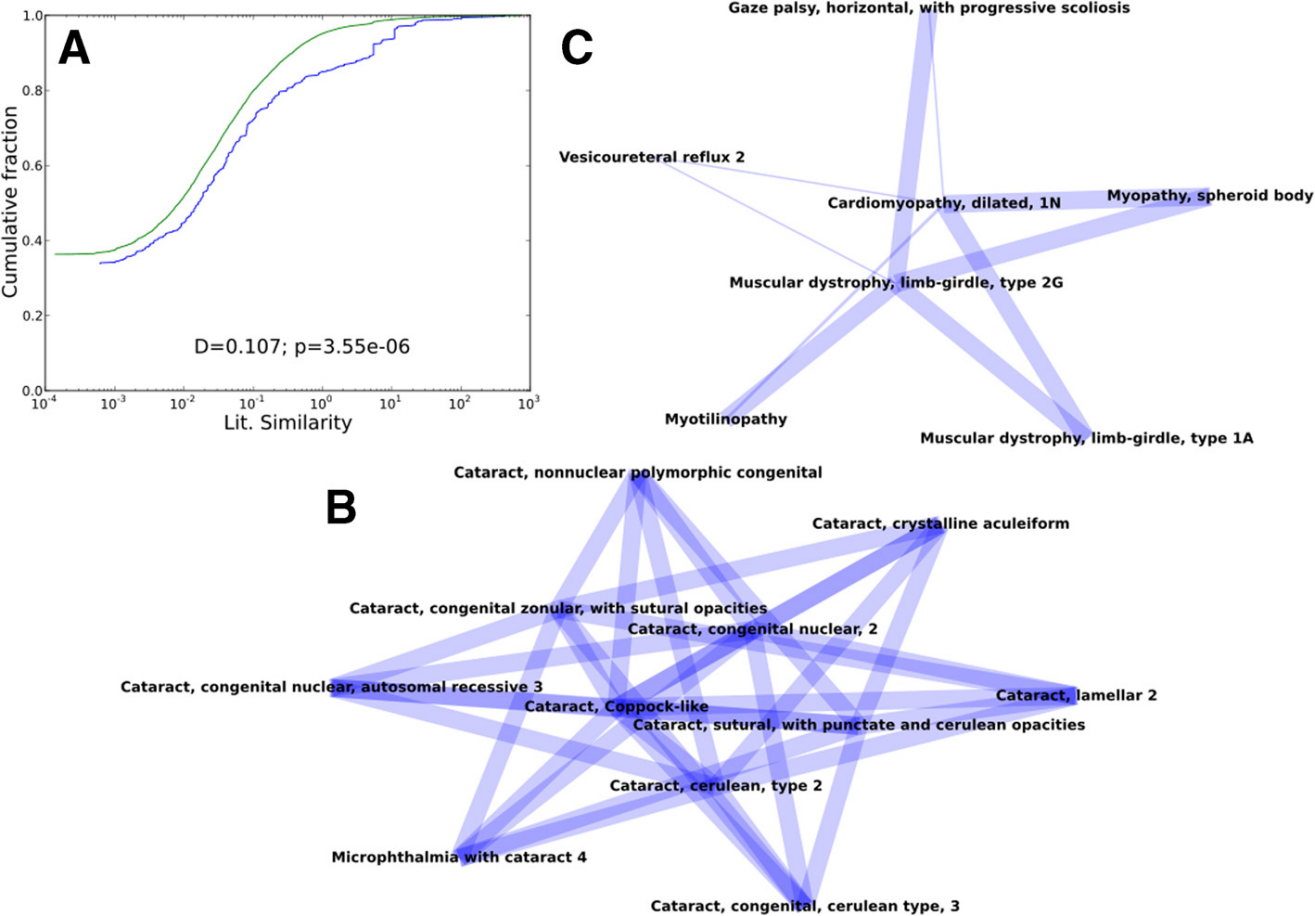
**OMIM human disease network.** **(A)** The cumulative distribution of literature similarity scores in randomized networks (green) and the observed human disease network constructed from ENTS PPI predictions (blue). **(B)** and **(C)** connected components in the human disease network. Line widths are drawn proportional to literature similarity scores.

As a demonstration of the potential for ENTs predictions to provide insights into human diseases, we created a network of associations between human diseases found in the OMIM database. Edges are inferred between diseases if the corresponding disease genes are predicted to produce interacting proteins within the ENTS 0.65 confidence human PPI network and if these interactions are more frequent than expected by random chance (see Methods). This leads to 552 disease associations covering 408 distinct pathologies and divided into 61 connected components (Additional file 4). Overall, we find the disease network to be significantly enriched for similarity in the literature relative to random networks (Kolmolgorov-Smirnov one-sided test; *D* = 0.0948; *p* = 5.065 × 10^−5^) (Figure 6A). The network contains a variety of intuitive and non-intuitive relationships between pathologies, many of which are based on interactions absent from the public databases.

As an example of intuitive relationships, one of the connected components consists entirely of associations between 11 cataract disorders (Figure 6B), all of which are based on predicted interactions between crystallin proteins without experimental support in public databases. We also find a number of non-intuitive relationships with anecdotal support in the literature. These include associations within one connected component containing several myopathies, including cardiomyopathy, limb-girdle muscular dystrophy, myotilinopathy, and spheroid body myopathy. These three latter disorders have overlapping symptoms and are known to co-occur with cardiomyopathy [25], as indicated by the network (Figure 6C). A disease characterized by progressive extraocular muscle weakness - horizontal gaze palsy with progressive scoliosis - is also associated with both cardiomyopathy and limb-girdle muscular dystrophy based on a predicted interaction between *TCAP* and *ROBO3*, though limb-girdle muscle weakness is not directly associated with this disorder in the literature [26]. The last condition within the component, vesicoureteral reflux (VUR), is characterized by developmental abnormalities of the kidney and urinary tract. Its relationship to cardiomyopathy and limb-girdle muscular dystrophy is indicated by a predicted interaction between *TCAP* and *ROBO2*. Interestingly, although VUR is not associated with disorders of the striated or cardiac muscle, it has been observed to co-occur with visceral myopathy in cases of Chronic Intestinal Pseudo Obstruction and Berdon Syndrome [27].

### Network properties and duplicate gene evolution

**Figure 7:**
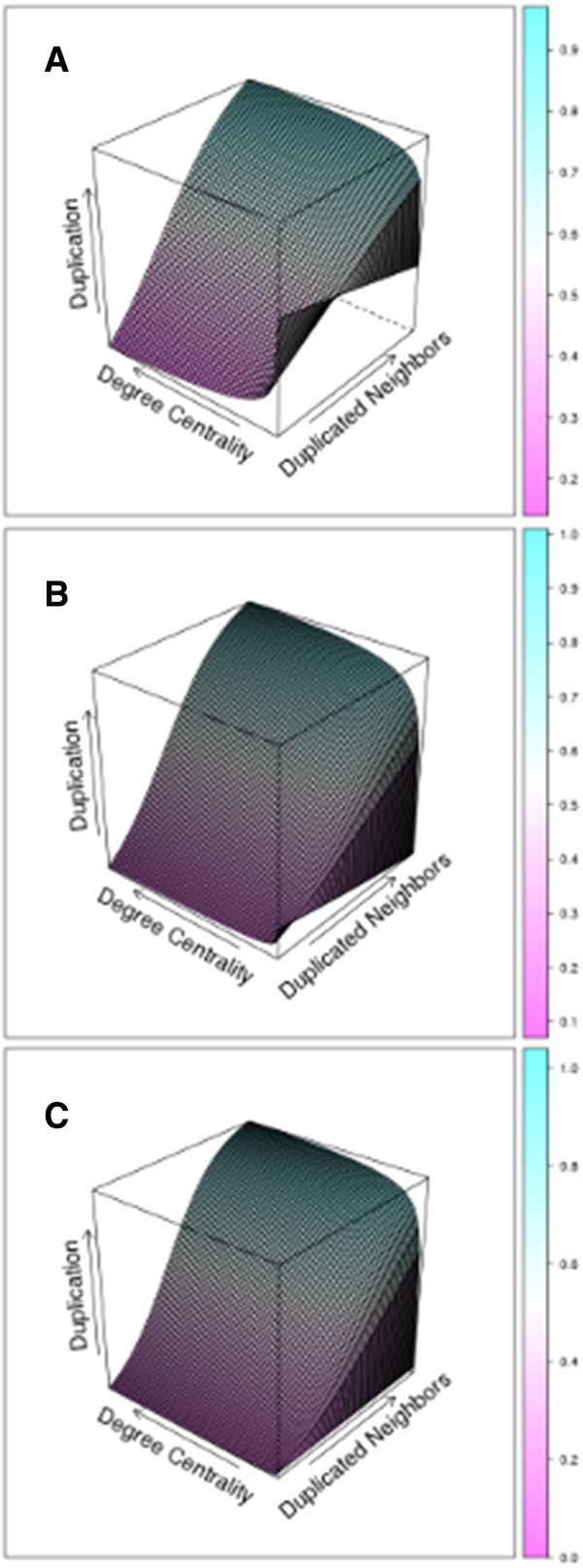
**Logistic models for duplicate retention based on ENTS predictions.** The relationship between degree centrality, the fraction of predicted neighbors retained following WGD, and the probability of whether a gene in the predicted network is retained following the WGD for the **(A)** *P. trichocarpa* Salicoid duplication, **(B)** the *A. thaliana* *α* duplication, and **(C)** the *A. thaliana* *β* and *γ* duplications.

Recently, several authors have proposed a relationship between the properties of biological networks and the evolution of duplicate genes. Studies of paleopolyploid plants have demonstrated that functional categories generally associated with higher network connectivity tend to be retained in duplicate following whole genome duplication (WGD) [28]. These observations led to the development of the gene balance hypothesis, which predicts that more highly connected genes should tend to be retained following WGD because of purifying selection for stoichiometric balance among interaction proteins [29]. Unfortunately, the lack of large-scale PPI data for paleopolyploid plants has largely precluded a thorough network-based analysis of this phenomenon. Here, we used the predicted *A. thaliana* and *P. trichocarpa* 0.65 confidence PPI networks to conduct a preliminary analysis of the relationship between WGD duplicate retention and 2 properties of the predicted network: the fraction of genes to which a given gene is connected (degree centrality) and the fraction of neighbors retained following the same WGD (duplicated neighbors). Based on logistic regression, the fraction of duplicated neighbors and the interaction term with degree centrality was positively associated with the presence of a duplicate paralog (Figure 7, Table 3). These results were highly consistent with those generated when we restricted the analysis to genes with at least 10 neighbors (Additional file 1: Table S3). Therefore, the duplication state of a given gene’s neighbors has a strong effect on its probability of retention, and this effect is enhanced with a higher number of interactions. This fits the predictions of the gene balance hypothesis in that the dependence on connectedness strongly depends upon the dosage of the interacting genes. Interestingly, the degree centrality main effect is negatively associated with the odds of retention once the interaction term is taken into account, although this effect is not significant for the *A. thaliana* *β*/*γ* WGD and inconsistently significant for the *α* WGD.

**Table 3:** Duplicate retention coefficients

**WGD**	**Intercept**	**log(DC)**	**DN**	**log(DC):DN**
*P. trichocarpa* Salicoid	-1.90193 ***	-0.24628 ***	4.91163 ***	0.57953 ***
*A. thalianaα*	-1.98648 ***	-0.09509 ***	5.73056 ***	0.69114 ***
*A. thalianaβ*/*γ*	-2.49904 ***	-0.03628	6.93515 ***	0.72433 ***

Estimated coefficients and significance for the logistic regressions on WGD retention in *P. trichocarpa* and *A. thaliana* using network properties degree centrality (DC) and duplicated neighbors (DN) from the ENTS 0.65 confidence networks. P-values are based on confidence intervals generated from fitting the model on resampled data 10,000 times.

*p < 0.05; **p < 0.01; ***p < 0.001.

## Discussion

### ENTS performance

In this study we find that our random forest-based classifier, ENTS, can predict biologically meaningful PPIs both within the species on which we trained the classifier and within species sharing a relatively distant common ancestor with the training species. These results are comparable or favorable to existing methods of protein interaction prediction, including several that used experimentally-obtained functional data as predictors. This demonstrates that researchers may generate a high quality set of probable PPIs prior to performing extensive functional experimentation. Therefore these predicted PPIs may serve as a basis for the development of functional hypotheses in newly-sequence organisms. Notably, even in the cases in which ENTS predictions were outperformed - the *A. thaliana* PAIR predictor and the *M. musculus* Yellaboina interolog approach - the majority of interactions predicted by ENTS were not predicted by the alternative approach. This suggests the value of using ENTS as part of an ensemble rather than relying on any single classifier. This may be particularly effective when large amounts of functional data are available, as with PAIR, or an orthologous interactome is well-covered, as with the Yellaboina interolog approach.

The performance of ENTS does vary significantly between organisms, with the AUC ranging from a low of 0.811 in *S. cerevisiae* to a high of 0.963 in *A. thaliana*. These differences likely arise due to functional differences between the positive datasets. The contrast in performance between the *A. thaliana* and non-plant classifiers may be attributed to biases in the interaction data currently available for these organisms - particularly *A. thaliana*. With the exception of a recent high-throughput study that was not used for training [5], *A. thaliana* studies of PPIs within the literature have focused on testing of specific hypotheses concerning proteins of high *a priori* importance. This can result in sets of highly clustered, high degree nodes within literature-curated PPI networks [5,30]. Such clusters have likely led to an enrichment of *A. thaliana* interacting pairs with high pairwise domain odds (Additional file 1: Figure S1). It also explains the decline in relative performance for *A. thaliana* predictions on proteins that were never used for training or calculation of pairwise domain odds (Additional file 1: Figure S2). In contrast, the multiple high throughput studies of binary PPIs conducted on *H. sapiens*[2], *S. cerevisiae*[3], and *D. melanogaster*[4] contribute a more unbiased view of the interactome than for *A. thaliana*. The more comprehensive experimental datasets have yielded a number of interactions that are less amenable to ENTS detection due to either an absence of PFAM domains in one or both of the proteins in the pair or because of the presence of domain pairs infrequently associated with physical interaction. Testing performance based primarily on literature-curated sets may therefore give results that are inconsistent with the true global protein interaction network. This highlights a necessary caveat present for all statistical learning methods - the performance of the classifier on novel data depends on the scope of its training.

Although the most important features for ENTS classification are based on domain composition, we also find subcellular localization information from MultiLoc2 to be highly important for prediction of PPIs. Interestingly, the most important MultiLoc2 features tend to be the SVM amino acid scores, which indicate how well the total amino acid content of a protein matches a particular subcellular compartment [31]. These measures provided ENTS with more sensitivity to subtle changes in amino acid content than the domain-based scores, which rely on the gain or loss of conserved PFAM domains. For instance, the duplicated genes from the Salicoid WGD in *P. trichocarpa* only share 56% of their predicted neighbors on average in the 0.65 confidence network despite high pairwise similarity in domain content. This allows for the possibility that ENTS may predict rewiring of PPIs within networks following duplication, although confirmation of this would require experimental validation.

### Interpretation of ENTS interactions

We trained ENTS using sets of known physically-interacting pairs of proteins. Therefore, proteins predicted to interact by ENTS should be interpreted as having a high potential for physical interaction, given that they are present within the same location at the same time. Each set of predicted interactions represents a more than 1000-fold reduction in the number of total possible interactions for the organism. However, because we want to permit the use of this classifier across a variety of organisms without broad functional data, we do not include expression or proteomics data as features in the set of predictor variables. As such, researchers should confirm all predictions with functional data. Furthermore, ENTS has limited capability for predicting interactions between proteins that either lack conserved domains or that contain domains never before experimentally observed within physically interacting proteins. This precludes the detection of some novel interactions, although other researchers may wish to append functional data to the set of ENTS predictor variables for their organism in order to predict these interactions through a greater variety of evidence sources.

### Applications of ENTS networks

The elucidation of PPI networks permits tremendous insight into both cellular and evolutionary processes. In humans, several studies have used experimental PPI data to map relationships between human diseases based on causative similarity and infer causative disease genes within large implicated linkage regions [32]. However, such studies have reported limitations due to the lack of detected interactions with shared disease etiology. Here, we have shown that ENTS is capable of yielding novel predicted interactions with relevance to known co-occurring human diseases. We contend that these predicted interactions may be used by biomedical researchers to narrow the scope of regions implicated in genome-wide association studies in addition to providing predictions on which to base more targeted searches for candidate loci. The latter function may prove especially vital due to the increasingly visible role of rare *de novo* mutations, particularly CNVs, in the etiology of disease [33]. Furthermore, the generality of our approach across species allows the potential for yielding insight into a variety of agriculturally important diseases in non-model species that are currently only understood through large linkage regions based on QTL and relatively small GWAS studies. We have also shown that ENTS is capable of revealing the higher-order structure of metabolic networks in a plant species without extensive experimental data, *P. trichocarpa*. ENTS-predicted physical interactions yield a significant enrichment of associations between pathways that share compounds and group sets of coordinated pathways such as pyrimidine and arginine biosynthesis [23]. Such insights may be used to inform targets of selection in breeding programs in order to increase the output flux from key pathways.

We have demonstrated that ENTS has great potential for yielding insight into network evolution in non-model species across the plant, animal, and fungal kingdoms. Networks have come to play an increasingly central role in evolutionary studies [6], but a rigorous analysis of network evolution requires the development of whole-genome networks for many non-model species. Here, we have shown that the topological properties of ENTS-predicted networks, including the first whole-genome interactome for *P. trichocarpa*, are related to the probability of WGD duplicate gene retention following independent duplication events in a manner consistent with the predictions of the gene balance hypothesis. Specifically, we have shown that the duplication state of a gene’s neighbors has a strong impact on the probability of retention following WGD and that this effect is enhanced at higher connectivity, which is consistent with the hypothesis of a selective drive to maintain stoichiometric balance between interacting proteins. Interestingly, the degree centrality main effect was only significant for the *P. trichocarpa* Salicoid WGD and the *A. thalianaα* WGD. In the case of *P. trichocarpa*, the negative degree centrality coefficient leads to an inverse relationship between connectivity and the probability of duplicate retention in the presence of few duplicated neighbors. This decrease is, again, consistent with the gene balance hypothesis, as the effects of stoichiometric imbalance are likely to become more extreme at higher connectivity and would therefore favor a singleton state when interconnected genes are also singletons. Intriguingly, this effect is also present in the *A. thaliana* duplications, though it is highly diminished with respect to *P trichocarpa* and shows inconsistent statistical significance. One explanation is that *A. thaliana* has undergone a greater degree of fractionation following its last two WGDs than has *P. trichocarpa* following the Salicoid WGD [34], so the influence of connectivity is more apparent in the latter. This suggests that the *P. trichocarpa* duplicate genes most at risk for future nonfunctionalization include those with low degree centrality and few duplicated neighbors.

### Conclusions

We have introduced an efficient new approach that enables prediction of protein-protein interactions on a whole genome scale based entirely on information that can be derived from primary sequence data. This is a potentially groundbreaking addition to the standard toolbox for newly-sequence non-model genomes, which are rapidly proliferating. The networks derived from our protein-protein interaction predictions are realistic from the standpoint of consistency with co-expression and shared functional annotations of connected genes. Furthermore, we have shown that our predictions can reveal supported relationships among emergent phenotypes such as human disease states and the coordination of metabolic pathways. Finally, we have demonstrated that our inferred networks can reveal subtle details of genome-scale evolution. Because the method can readily be applied on a large scale to phylogenetically-diverse organisms, we anticipate that large-scale comparative analyses will provide insights into the mechanisms of network structure evolution.

## Methods

### Data sources

We obtained experimentally verified physical interactions for *H. sapiens*, *S. cerevisiae*, *M. musculus*, *D. melanogaster*, and *A. thaliana* from the PPI databases DIP (10/27/2011 Release) [35], IntAct (01/01/2012 Release) [36], and BioGRID (v. 3.1.84) [37]. Additional experimentally-verified interactions for *A. thaliana* were taken from the TAIR database (05/27/2009 Release) [38]. We also collected known and predicted domain-domain interactions and their associated confidence scores from the DOMINE database (version 2) [11]. All protein annotations were taken from Ensembl core databases with the *A. thaliana* and *P. trichocarpa* annotations corresponding to Ensembl Plants release 12, the *S. cerevisiae* annotations corresponding to Ensembl Fungi release 12, and the *H. sapiens*, *M. musculus*, and *D. melanogaster* annotations corresponding to Ensembl release 65. Only canonical versions of proteins were used; splice variants were not considered in this analysis.

### Calculation of domain pair odds

We calculated a log-of-odds score for each pair of domains observed at least once in an interacting protein pair. This score may be interpreted as the odds of observing a pair of domains in an interacting protein pair versus by random chance among all interacting proteins. We obtained all unique PFAM domains present in each protein for all experimentally-verified protein interactions in *H. sapiens*, *S. cerevisiae*, *M. musculus*, *D. melanogaster*, and *A. thaliana* with the exception of 1,300 in each training organism that were reserved for testing data. We then assessed all possible pairwise domain combinations among all these protein pairs and calculated the log-odds score for each domain pair as follows:

(1) $$\begin{array}{l} f(D_{x},D_{y})=\frac{n(D_{x},D_{y})}{\sum_{i=1}^{n_{p}}\sum_{j=1}^{i}n(D_{i},D_{j})}\; \end{array}$$

(2) $$\begin{array}{l} f(D_{x})=\frac{n(D_{x})}{\sum_{i}n(D_{i})}\; \end{array}$$

(3) $$\begin{array}{l} \text{LOD}=\log\frac{f(D_{x},D_{y})}{f(D_{x})f(D_{y})}\; \end{array}$$

where *n*(*D*_*x*_,*D*_*y*_) is the number of times the domain pair *D*_*x*_, *D*_*y*_ was observed among experimentally-verified protein interactions, *n*_*p*_ is the total number of domain pairs, and *n*(*D*_*x*_) is the number of times *D*_*x*_ was observed among the set of proteins with experimentally-verified interactions. Because the absence of a protein pair within the experimentally-verified data set may result from either non-detection of existing interactions or the absence of any such interactions, we chose to assume an LOD of 0 for all domain pairs that were not observed in any interacting protein pairs.

### Feature data

All data features were defined from pairwise-domain information and predictions of subcellular localization. An exhaustive list of all features can be found in Additional file 1: Table S1. Domain-based features included the sum of all odds scores, the highest odds score, the lowest odds score (ceilinged at 0), the number of pairwise domain pairs not observed in any of the interacting proteins used to calculate the odds scores, the number of domain pairs found among the pairs with odds scores, the number of domain pairs predicted or known to interact in DOMINE, and the highest DOMINE confidence score assigned to a domain pair. All other features were outputs of the high-res MultiLoc2 subcellular predicted program [31], which used protein sequence data and computer-generated GO categories as input. These features included the probabilities of localization to each of the possible subcompartments - cytoplasm, nucleus, peroxisome, ER, mitochondria, chloroplast (plant only), vacuole (plant and fungus), and the lysosome (animal only) - along with the raw output from each of the MultiLoc2 subprograms - SVMTarget, SVMSA, SVMaac, PhyloLoc, GOLoc, and MotifSearch.

### Training and prediction

Classifiers were trained using 1,330 randomly selected protein pairs with experimental verification of interaction to serve as positive examples for each organism. We also included 101,300 randomly drawn pairs of proteins without any known or predicted interactions in the Reactome version 39 database to serve as negative examples. In order to avoid potential over-fitting based on the predicted subcellular characteristics of proteins in the positive set, we spiked the negative set with 1,300 randomly selected pairs of proteins without known or predicted interactions drawn from the positive set, wherein the proteins in the pair were drawn in proportion to their representation within the interacting pairs. Additionally, we included each protein pair twice within the dataset but switched the subcellular features between the proteins in the second set (reversed set). We trained the classifier using 400 fully-grown trees with the R randomForest package ( http://cran.r-project.org/web/packages/randomForest/). Prediction also used both the forward and reversed set, with the final predictions taken from the union of the two. We trained a total of 3 ENTS classifiers - one on *H. sapiens*, one on *S. cerevisiae*, and one on *A. thaliana*. The *H. sapiens*-trained classifier was used to predict on *H. sapiens* and *M. musculus*; the *S. cerevisiae*-trained classifier was used to predict on *S. cerevisiae*; and the *A. thaliana*-trained classifier was used to predict on *A. thaliana* and *P. trichocarpa*.

In order to characterize the testing performance, we calculated the area under the ROC curve (AUC) using the R pROC package ( http://cran.r-project.org/web/packages/pROC/index.html). Testing data consisted of 1,300 known positive interactions and 101,300 randomly selected negative examples without known or predicted interactions. Testing data did not include any protein pairs used to calculate the domain pair odds and contained no overlap to the random forest training data at the level of protein interactions. Sensitivity and specificity are defined as follows:

(4) $$\text{sensitivity}=\frac{\text{TP}}{\text{TP}+\text{FN}}$$

(5) $$\text{specificity}=\frac{\text{TN}}{\text{TN}+\text{FP}}$$

where *TP* is the number of true positives (predicted interactions with experimental support), *FN* is the number of false negatives (non-predicted interactions with experimental support, *TN* is the number of true negatives (non-predicted interactions without experimental support), and *FP* is the number of false positives (predicted interactions without experimental support). We also calculated these values for the other predictors using the same set of testing data to provide single points on the ROC curve.

### Comparisons to other predictors

Predicted protein interaction datasets were taken from the Pitre *et al.* PIPE2 *S. cerevisiae* classifier novel interactions [14], the *H. sapiens* PIPS classifier [15], the NIA Mouse Protein-Protein Interaction Database [16], the predicted *A. thaliana* interactome of De Bodt *et al.*[7], the AtPID *A. thaliana* interactome [8], the Geisler-Lee *A. thaliana* interactome [9], and the PAIR high-confidence *A. thaliana* interactome [10]. We obtained protein interaction datasets without any overlap to the interactions used to train ENTS or calculate domain odds scores. We also limited the overlap to training data from the alternative classifiers by choosing datasets containing PPIs that were either published after the alternative classifier’s publication date or were not present within the datasets reportedly used for training. In *S. cerevisiae* these were taken from a high-throughput yeast two-hybrid study [3]. In humans we used high confidence sets of human protein interactions from a recent study of protein complexes (confidence of at least 0.9) [19] and a high-throughput mass-spectrometry study of human PPIs (confidence of at least 0.3) [18]. In *M. musculus* we used interactions taken from the HitPredict database (05/01/2012) that were published after October 2008 [39]. Finally, in *A. thaliana* we used high-throughput yeast two-hybrid and literature curated interactions from a large study of network evolution [5].

Prior to performing each comparison we removed any predicted pairs from ENTS and the alternative classifiers that were used for training of the ENTS random forest or calculation of domain pair odds. ENTS predictions were then sorted by decreasing confidence and truncated to sets of the same size as the alternative classifiers. We then calculated the sizes of the intersections with the experimental datasets in addition to the sizes of the three-way intersections between ENTS, the alternative classifiers, and the experimental datasets. We then performed the same comparisons after limiting the predicted datasets to pairs of proteins in which neither protein was present within the training data or the proteins used to calculate domain-pair odds.

### Evaluation of functional similarity

For each measure of functional similarity, we first narrowed the protein network (predicted or experimentally-verified) to the set of proteins with the given annotation present. For instance, we narrowed the networks to those with at least 1 KEGG annotation when evaluating KEGG similarity. Similarity was then calculated as the number of pathways present in the intersection of two proteins’ annotations divided the by the number of pathways in the union.

GO semantic similarity between pairs of putatively interacting proteins was calculated separately for biological process, cellular component, and molecular function, and was based on the information content of shared parents [40]. Briefly, for each organism we assigned a probability to each GO subgraph node, *p*(*c*), which was defined as follows:

(6) $$p(x)=\frac{n(c_{d})}{n_{c}}$$

where *n*(*c*_*d*_) is the number of times the node or any of its descendants occurred in the genome, and *n*_*c*_ is the number of times any term occurred. For each pair of queried terms we then found the shared parent with the minimal probability *p*_*m*_(*c*_*x*_,*c*_*y*_) among the set of shared parents in the subgraph, *S*(*c*_*x*_,*c*_*y*_):

(7) $$p_{m}(c_{x},c_{y})=\underset{c\in s(c_{x},c_{y})}{\text{min}}p(c)$$

The similarity score between the two terms *c*_*x*_ and *c*_*y*_ was then defined as follows:

(8) $$\text{sim}(c_{x},c_{y})=-\text{ln}(p_{m}(c_{x},c_{y}))$$

In the case of more than one pair of GO terms between a pair of proteins, we set the similarity score as the maximum of all pairwise similarity scores.

### Metabolic pathway linkages

We made a network of linkages between metabolic pathways downloaded from the PoplarCyc v. 3.0 database [21] using protein interactions predicted in the *P. trichocarpa* 0.65 confidence network. Pathways were linked to one another if one or more protein pairs between the two pathways were predicted to interact. We also did not include predicted self-interactions when constructing the network. We tested the significance of all pathway linkages by creating 10,000 randomized PPI networks in which each node had the same degree as the ENTS network but randomized connections. We retained pathway linkages for which the number of supporting interactions was greater than that of at least 99.9% of randomized networks. We found the number of pathway linkages that connected two pathways sharing at least one metabolic compound between them, discounting all compounds that were present in more than 15 pathways in order to prevent less meaningful associations due to common compounds such as ATP. We then assessed the significance of the compound sharing by generating 10,000 randomized pathway linkage networks in which each pathway had the same degree as in the observed network.

We performed clustering on the pathway linkage network using the MCL graph clustering algorithm with default parameters [41]. Edges were weighted by the fraction of interactions predicted to exist between the two pathways out of the total number of possible non-self interactions. We then tested the clusters for significant enrichment using annotation enrichment analysis, which corrects for biases that can occur under Fisher’s exact test [42]. Briefly, each pathway was contained within a pathway ontology retrieved from PlantCyc, forming a directed acyclic graph. Each node in the graph was annotated with a given pathway if that pathway was a descendant of the node. P-values were then generated for each term in the ontology as follows:

(9) p(Mgt)=∑i=Mgtmin(Mg,Mt)MtiMtot−MtMg−iMtotMg

where *M*_*g*_ is the number of ontology annotations to the cluster, *M*_*gt*_ is the number of ontology annotations to the cluster on the ontology branch of interest, *M*_*t*_ is the number of pathways annotated to the branch of interest, and *M*_*tot*_ is the total number of pathway annotations made to the ontology graph. We assessed significance using a 0.05 family-wide type I error rate under a Bonferroni correction, such that a term was considered significant if $p\leq \frac{0.05}{n_{C}n_{O}}$ where *n*_*C*_ is the number of clusters found and *n*_*O*_ is the number of nodes in the ontology.

### OMIM disease network

We created a network of OMIM diseases and disorders by creating an edge between two diseases if their underlying causative loci in the OMIM database were predicted to interact within the ENTS predicted human network. We did not count self-interactions when creating the network. We tested the significance of the disease-disease associations by creating 10,000 randomized PPI networks in which each node had the same degree as the ENTS network but randomized connections. We then retained disease associations for which the number of supporting interactions was greater than that of at least 99.9% of randomized networks.

We then evaluated the significance of the network associations using Pubmed literature mining. In order to perform this with a controlled vocabulary, we first mapped the OMIM identifiers for each disease to medical subject headings (MeSH) terms using the Gendoo database [43], narrowing the disease network to OMIM identifiers associated with at least 1 MeSH term. Each of these OMIM-MeSH associations was associated with a p-value indicating the significance of the term-term association, *p*_*OM*_. We obtained the full set of Pubmed IDs (PMIDs) associated with each MeSH term present within the disease network using NCBI E-Utilities. We then assessed literature similarity using a measure that incorporated both the significance of the OMIM-MeSH association and the frequency of the MeSH-MeSH pairings within the literature. This was calculated as follows:

(10) $$\begin{array}{ll} \;\text{sim}(M_{x},M_{y}) & =-\frac{n(P_{x}\cap P_{y})}{\text{min}(n(P_{x}),n(P_{y}))}\\ & \;\times \log(\text{max}(p_{\text{OM}}(M_{x}),p_{\text{OM}}(M_{y}))) \end{array}$$

where the $n(\dot{)}$ function refers to the number of PMIDs in the set, and *P*_*x*_, *P*_*y*_ are the sets of PMID ids associated with the MeSH terms *M*_*x*_ and *M*_*y*_. Only the most significant MeSH term for each OMIM disease was used for comparison. In order to assess the significance of the observed network, we compared the distribution of literature scores to those in 250 random disease-disease networks in which disease nodes were sampled in proportion to their degree in the observed network, thus generating networks with similar degree distributions.

### WGD logistic regression

Intragenomic syntenic segments corresponding to the Salicoid WGD in *P. trichocarpa* and the *α* and *β*/*γ* duplications in *A. thaliana* were defined using MCScan [44]. We separated the more recent *α*-duplication from the older *β* and *γ* duplications using the mappings provided by Bowers *et al* (2003) [45]. However, many of the duplicated regions with higher dS were not defined within that dataset, and we could not confidently separate the *β* and *γ* duplications from one another based on dS due to saturation of substitutions. Therefore, we considered the *A. thaliana* *β* and *γ* WGDs as a single *β*/*γ* duplication for the purposes of the analysis. We then fit the following model using the generalized linear model with the logit link function in the R programming language:

(11) $$\begin{array}{ll} \;\text{Duplication} & ∼\text{log(Degree Centrality)}+\text{Duplicated Neighbors}\\ & \;+\;\text{log(Degree Centrality)}\;:\;\text{Duplicated Neighbors} \end{array}$$

The response was coded as 0 or 1, corresponding to absence or presence of a duplicate paralog from the given WGD, respectively. The duplicated neighbors term was calculated as the fraction of neighboring genes that were retained as duplicates from the corresponding WGD. Degree centrality was calculated as follows using the Python Networkx package ( http://networkx.lanl.gov):

(12) $$\text{Degree Centrality}=\frac{d(n_{x})}{n_{G}-1}$$

where *n*_*G*_ is the number of nodes in the graph, and *d*(*n*_*x*_) is the degree of node *n*_*x*_. We assessed the significance of the model coefficients by resampling 10,000 times with replacement, fitting the model to the resampled data, and generating 95%, 99%, and 99.9% confidence intervals using the corresponding quantiles of the coefficient distributions. Additionally, because the degree centrality constrained the possible range of the duplicated neighbor fraction such that a gene with one neighbor could only have a duplicated neighbor value of either 0 or 1, we fit two models for each duplication: one using the full set of genes with at least 1 interaction in the predicted network and one using only the subset with at least 10 predicted interactions.

## Implementation

ENTS is implemented using a combination of Python and R. The user provides a tab-delimited list of proteins and their constituent PFAM domains along with high-resolution output from the subcellular localization prediction program Multiloc2. The user also specifies a set of two tab-delimited files. The first file contains the pairwise domain odds for pairs of PFAM domains potentially involved in interactions, and the second provides PFAM pairwise scores from the DOMINE database. We provide both of these flat files for convenience, along with R workspaces with random forests trained on *A. thaliana*, *H. sapiens*, and *S. cerevisiae*.

ENTS is run by calling a Python script from the command line. The Python script then splits the $\frac{n(n+1)}{2}$ pairwise comparisons to be performed among a number of subprocesses specified by the user at the command line. These are run in parallel, and each subprocess makes calls to the random forest present in the R workspace through Rserve using the pyRserve package as an interface. The random forest is implemented using the efficient R randomForest package. Protein pairs with confidence scores above a user-specified threshold are then saved to tab-delimited files, which are combined into a single file at the end of the run.

## Availability and requirements

Project Name: ENTS

Home Page: http://ents.as.wvu.edu

Operating system(s): Windows, Unix-like (Linux, Mac OSX)

Programming language: Python >= 2.7, R >= 2.15

Dependencies: Python - Numpy and pyRserve, R - randomForest and Rserve, MultiLoc2 (Optional)

## Competing interests

The authors declare that they have no competing interests.

## Authors’ contributions

ERM and SD conceived the study and wrote the manuscript. ERM wrote the the source code and made all figures. MC provided advice on statistical methods. All authors read and approved the final manuscript.

## Supplementary Material

Additional file 1 **Supplementary Figures and Tables.** PDF format, containing additional ENTS performance measures. Click here for file

Additional file 2 ***P. trichocarpa* metabolic pathway linkage network.** Tab-delimited file containing the weighted pathway linkages in the PoplarCyc v. 3 metabolic network. Click here for file

Additional file 3 ***P. trichocarpa* metabolic pathway linkage network clusters.** Tab-delimited file containing the clusters of pathways found by the MCL algorithm. Click here for file

Additional file 4 **Human disease association network.** Tab-delimited file containing the associations discovered between OMIM diseases and the numbers of predicted interactions supporting those associations. Click here for file
