# Supplementary material for: Predicting whole genome protein interaction networks from primary sequence data in model and non-model organisms using ENTS
Source: BMC Genomics. 2013 Sep 10;14:608. doi: 10.1186/1471-2164-14-608 (PMC3848842; doi:10.1186/1471-2164-14-608)
Supplement: Additional file 1 — Supplementary Figures and Tables. PDF format, containing additional ENTS performance measures. [file 1471-2164-14-608-S1.pdf]

Supplemental Table 1. Predictors used for the ENTS classifier. Importance measures indicate the mean decrease in accuracy in the out-of-bag training data upon permutation of that variable.

**Feature**	**Description**	***A. thaliana*** **Importance**	***S. cerevisae*** **Importance**	***H. sapiens*** **Importance**
sum_odds	Sum of the odds scores for unique pairs of domains in the protein pair	0.0621	0.0536	0.0521
highest_odds	The highest observed odds score for a pair of domains in the protein pair (floored at 0)	0.0604	0.0530	0.0578
lowest_odds	The lowest observed odds score for a pair of domains in the protein pair (capped at 0)	0.0500	0.0414	0.0313
not_observed	The number of unique domain pairs in the protein pair never observed in any known interactions	0.0573	0.0646	0.0627
not_observed_frac	The fraction of unique domain pairs in the protein pair never observed in any known interactions	0.0594	0.0470	0.0564
n_odds_pairs	The number of unique domain pairs observed in at least 1 known interaction	0.0441	0.0404	0.0435
n_domine_pairs	The number of unique domain pairs predicted or verified to interact in the DOMINE database	0.0370	0.0247	0.0342
highest_domine_conf	The highest confidence score for a pair of domains verified to interact in the DOMINE database	0.0432	0.0273	0.0389
cytoplasmic1	The MultiLoc2 probability of localization in the cytoplasm for the first protein in the pair	0.0313	0.0458	0.0445
nuclear1	The MultiLoc2 probability of localization in the nucleus for the first protein in the pair	0.0420	0.0480	0.0394
peroxisomal1	The MultiLoc2 probability of localization in the peroxisome for the first protein in the pair	0.0421	0.0367	0.0383
ER1	The MultiLoc2 probability of localization in the ER for the first protein in the pair	0.0305	0.0289	0.0336
mitochondrial1	The MultiLoc2 probability of localization in the mitochondrion for the first protein in the pair	0.0384	0.0352	0.0423
extracellular1	The MultiLoc2 probability of localization in the extracellular space for the first protein in the pair	0.0371	0.0278	0.0316
plasma membrane1	The MultiLoc2 probability of localization in the plasma membrane for the first protein in the pair	0.0306	0.0301	0.0326
Golgi apparatus1	The MultiLoc2 probability of localization in the Golgi apparatus for the first protein in the pair	0.0250	0.0355	0.0354
chloroplast1	The MultiLoc2 probability of localization in the chloroplast for the first protein in the pair (plant only)	0.0193	N/A	N/A
vacuolar1	The MultiLoc2 probability of localization in the vacuole for the first protein in the pair (plant and fungal)	0.0316	0.0237	N/A
lysosomal1	The MultiLoc2 probability of localization in the lysosome for the first protein in the pair (animal only)	N/A	N/A	0.0318
svm_target_score_sp1	MultiLoc2 score for detection of N-terminal targeting peptides for secretory pathways in the first protein of the pair	0.0439	0.0292	0.0433
svm_target_score_mtp1	MultiLoc2 score for detection of N-terminal targeting peptides for mitochondrion in the first protein of the pair	0.0300	0.0351	0.0390
svm_target_score_ctp1	MultiLoc2 score for detection of N-terminal targeting peptides for chloroplast in the first protein of the pair (plant only)	0.0183	N/A	N/A
svm_target_score_mtp_vs_ctp1	MultiLoc2 score for detection of N-terminal targeting peptides contrasting mitochondrion with chloroplast in the first protein of the pair (plant)	0.0395	N/A	N/A
svm_target_score_oth1	MultiLoc2 score for detection of N-terminal targeting peptides for other compartments in the first protein of	0.0252	0.0279	0.0367

	the pair			
svm_sa_score1	MultiLoc2 score for presence of signal anchor for membrane proteins of secretory pathway in the first protein of the pair	0.0438	0.0470	0.0384
svm_aac_score_nuc1	MultiLoc2 score for whether the overall amino acid content of the first protein matches the nucleus	0.0313	0.0464	0.0382
svm_aac_score_cyt1	MultiLoc2 score for whether the overall amino acid content of the first protein matches the cytoplasm	0.0409	0.0481	0.0497
svm_aac_score_nuc_vs_cyt1	MultiLoc2 score for contrasting the overall amino acid content of the first protein with respect to nuclear vs. cytoplasm localization	0.0234	0.0421	0.0318
svm_aac_score_mit1	MultiLoc2 score for whether the overall amino acid content of the first protein matches the mitochondrion	0.0474	0.0474	0.0501
svm_aac_score_chl1	MultiLoc2 score for whether the overall amino acid content of the first protein (plant) matches the chloroplast	0.0504	N/A	N/A
svm_aac_score_ext1	MultiLoc2 score for whether the overall amino acid content of the first protein matches the extracellular space	0.0503	0.0490	0.0503
svm_aac_score_pm1	MultiLoc2 score for whether the overall amino acid content of the first protein matches the plasma membrane	0.0508	0.0525	0.0544
svm_aac_score_per1	MultiLoc2 score for whether the overall amino acid content of the first protein matches the peroxisome	0.0420	0.0460	0.0522
svm_aac_score_er1	MultiLoc2 score for whether the overall amino acid content of the first protein matches the ER	0.0511	0.0409	0.0506
svm_aac_score_gol1	MultiLoc2 score for whether the overall amino acid content of the first protein matches the Golgi apparatus	0.0501	0.0512	0.0543
svm_aac_score_vac1	MultiLoc2 score for whether the overall amino acid content of the first protein matches the vacuole (plant and fungal)	0.0441	0.0442	N/A
goloc_score_nuc1	MultiLoc2 score for whether inferred GO categories of the first protein match the nucleus	0.0406	0.0344	0.0462
goloc_score_cyt1	MultiLoc2 score for whether inferred GO categories of the first protein match the cytoplasm	0.0370	0.0364	0.0397
goloc_score_mit1	MultiLoc2 score for whether inferred GO categories of the first protein match the mitochondrion	0.0381	0.0342	0.0404
goloc_score_chl1	MultiLoc2 score for whether inferred GO categories of the first protein match the chloroplast (plant)	0.0277	N/A	N/A
goloc_score_ext1	MultiLoc2 score for whether inferred GO categories of the first protein match the extracellular space	0.0307	0.0287	0.0470
goloc_score_pm1	MultiLoc2 score for whether inferred GO categories of the first protein match the plasma membrane	0.0356	0.0299	0.0441
goloc_score_per1	MultiLoc2 score for whether inferred GO categories of the first protein match the peroxisome	0.0433	0.0335	0.0387
goloc_score_er1	MultiLoc2 score for whether inferred GO categories of the first protein match the ER	0.0375	0.0312	0.0466
goloc_score_gol1	MultiLoc2 score for whether inferred GO categories of the first protein match the Golgi apparatus	0.0310	0.0333	0.0405
goloc_score_vac1	MultiLoc2 score for whether inferred GO categories of the first protein match the vacuole (plant and fungus)	0.0400	0.0372	N/A
phyloloc_score_nuc1	MultiLoc2 score for whether phylogenetic profile of first protein BLAST hits indicates nuclear localization	0.0426	0.0433	0.0484
phyloloc_score_cyt1	MultiLoc2 score for whether phylogenetic profile of first protein BLAST hits indicates cytoplasmic	0.0458	0.0468	0.0476

	localization			
phyloloc_score_mit1	MultiLoc2 score for whether phylogenetic profile of first protein BLAST hits indicates mitochondrial localization	0.0470	0.0560	0.0494
phyloloc_score_chl1	MultiLoc2 score for whether phylogenetic profile of first protein BLAST hits indicates chloroplast localization (plant)	0.0471	N/A	N/A
phyloloc_score_ext1	MultiLoc2 score for whether phylogenetic profile of first protein BLAST hits indicates extracellular localization	0.0521	0.0468	0.0468
phyloloc_score_pm1	MultiLoc2 score for whether phylogenetic profile of first protein BLAST hits indicates plasma membrane localization	0.0485	0.0508	0.0501
phyloloc_score_per1	MultiLoc2 score for whether phylogenetic profile of first protein BLAST hits indicates peroxisomal localization	0.0512	0.0533	0.0498
phyloloc_score_er1	MultiLoc2 score for whether phylogenetic profile of first protein BLAST hits indicates ER localization	0.0503	0.0537	0.0517
phyloloc_score_gol1	MultiLoc2 score for whether phylogenetic profile of first protein BLAST hits indicates Golgi apparatus localization	0.0446	0.0475	0.0511
phyloloc_score_vac1	MultiLoc2 score for whether phylogenetic profile of first protein BLAST hits indicates vacuolar localization (plant and fungal)	0.0507	0.0529	N/A
motif_search_er_target1	MultiLoc2 indicator of ER targeting signal for the first protein	0.0019	0.0011	0.0013
motif_search_pm_receptor_domain1	MultiLoc2 indicator of plama membrane targeting signal for the first protein	0.0000	-0.0079	0.0166
motif_search_peroxi_target1	MultiLoc2 indicator of peroxisomal targeting signal for the first protein	0.0092	-0.0032	0.0101
motif_search_nuclear_bipartite1	MultiLoc2 indicator of nuclear bitartite protein for the first protein	0.0118	0.0111	0.0111
motif_search_predictNLS1	MultiLoc2 indicator of nuclear localization signal for the first protein	0.0144	0.0138	0.0150
motif_search_nls_mono1	MultiLoc2 indicator of nuclear localization signal for the first protein	0.0160	0.0219	0.0243
motif_search_dna_associated_domain1	MultiLoc2 indicator of dna associated domain for the first protein	0.0183	0.0062	0.0253
phyloloc_score_lys1	MultiLoc2 score for whether phylogenetic profile of first protein BLAST hits indicates lysosome localization (animal)	N/A	N/A	0.0547
goloc_score_lys1	MultiLoc2 score for whether inferred GO categories of the first protein match the lysosome membrane (animal)	N/A	N/A	0.0422
svm_aac_score_lys1	MultiLoc2 score for whether the overall amino acid content of the first protein matches the lysosome (animal)	N/A	N/A	0.0507
cytoplasmic2	The MultiLoc2 probability of localization in the cytoplasm for the first protein in the pair	0.0383	0.0410	0.0403
nuclear2	The MultiLoc2 probability of localization in the nucleus for the second protein in the pair	0.0455	0.0486	0.0364
peroxisomal2	The MultiLoc2 probability of localization in the peroxisome for the second protein in the pair	0.0418	0.0379	0.0442
ER2	The MultiLoc2 probability of localization in the ER for the second protein in the pair	0.0323	0.0211	0.0276
mitochondrial2	The MultiLoc2 probability of localization in the mitochondrion for the second protein in the pair	0.0315	0.0403	0.0312

extracellular2	The MultiLoc2 probability of localization in the extracellular space for the second protein in the pair	0.0372	0.0323	0.0344
plasma membrane2	The MultiLoc2 probability of localization in the plasma membrane for the second protein in the pair	0.0352	0.0329	0.0371
Golgi apparatus2	The MultiLoc2 probability of localization in the Golgi apparatus for the second protein in the pair	0.0307	0.0364	0.0357
chloroplast2	The MultiLoc2 probability of localization in the chloroplast for the second protein in the pair (plant only)	0.0255	N/A	N/A
vacuolar2	The MultiLoc2 probability of localization in the vacuole for the second protein in the pair (plant and fungal)	0.0344	0.0283	N/A
lysosomal2	The MultiLoc2 probability of localization in the lysosome for the second protein in the pair (animal only)	N/A	N/A	0.0303
svm_target_score_sp2	MultiLoc2 score for detection of N-terminal targeting peptides for secretory pathways in the second protein of the pair	0.0442	0.0374	0.0431
svm_target_score_mtp2	MultiLoc2 score for detection of N-terminal targeting peptides for mitochondrion in the second protein of the pair	0.0326	0.0366	0.0357
svm_target_score_ctp2	MultiLoc2 score for detection of N-terminal targeting peptides for chloroplast in the second protein of the pair (plant only)	0.0314	N/A	N/A
svm_target_score_mtp_vs_ctp2	MultiLoc2 score for detection of N-terminal targeting peptides contrasting mitochondrion with chloroplast in the second protein of the pair (plant)	0.0442	N/A	N/A
svm_target_score_oth2	MultiLoc2 score for detection of N-terminal targeting peptides for other compartments in the second protein of the pair	0.0367	0.0375	0.0397
svm_sa_score2	MultiLoc2 score for presence of signal anchor for membrane proteins of secretory pathway in the second protein of the pair	0.0436	0.0423	0.0396
svm_aac_score_nuc2	MultiLoc2 score for whether the overall amino acid content of the second protein matches the nucleus	0.0346	0.0411	0.0445
svm_aac_score_cyt2	MultiLoc2 score for whether the overall amino acid content of the second protein matches the cytoplasm	0.0253	0.0504	0.0505
svm_aac_score_nuc_vs_cyt2	MultiLoc2 score for contrasting the overall amino acid content of the second protein with respect to nuclear vs. cytoplasm localization	0.0281	0.0349	0.0380
svm_aac_score_mit2	MultiLoc2 score for whether the overall amino acid content of the second protein matches the mitochondrion	0.0513	0.0480	0.0551
svm_aac_score_chl2	MultiLoc2 score for whether the overall amino acid content of the second protein (plant) matches the chloroplast	0.0522	N/A	N/A
svm_aac_score_ext2	MultiLoc2 score for whether the overall amino acid content of the second protein matches the extracellular space	0.0521	0.0542	0.0537
svm_aac_score_pm2	MultiLoc2 score for whether the overall amino acid content of the second protein matches the plasma membrane	0.0508	0.0528	0.0555
svm_aac_score_per2	MultiLoc2 score for whether the overall amino acid content of the second protein matches the peroxisome	0.0485	0.0420	0.0536
svm_aac_score_er2	MultiLoc2 score for whether the overall amino acid content of the second protein matches the ER	0.0474	0.0425	0.0477
svm_aac_score_gol2	MultiLoc2 score for whether the overall amino acid	0.0454	0.0506	0.0519

	content of the second protein matches the Golgi apparatus			
svm_aac_score_vac2	MultiLoc2 score for whether the overall amino acid content of the second protein matches the vacuole (plant and fungal)	0.0458	0.0434	N/A
goloc_score_nuc2	MultiLoc2 score for whether inferred GO categories of the second protein match the nucleus	0.0377	0.0322	0.0440
goloc_score_cyt2	MultiLoc2 score for whether inferred GO categories of the second protein match the cytoplasm	0.0413	0.0308	0.0337
goloc_score_mit2	MultiLoc2 score for whether inferred GO categories of the second protein match the mitochondrion	0.0380	0.0328	0.0388
goloc_score_chl2	MultiLoc2 score for whether inferred GO categories of the second protein match the chloroplast (plant)	0.0380	N/A	N/A
goloc_score_ext2	MultiLoc2 score for whether inferred GO categories of the second protein match the extracellular space	0.0386	0.0283	0.0436
goloc_score_pm2	MultiLoc2 score for whether inferred GO categories of the second protein match the plasma membrane	0.0392	0.0352	0.0435
goloc_score_per2	MultiLoc2 score for whether inferred GO categories of the second protein match the peroxisome	0.0368	0.0357	0.0365
goloc_score_er2	MultiLoc2 score for whether inferred GO categories of the second protein match the ER	0.0315	0.0299	0.0392
goloc_score_gol2	MultiLoc2 score for whether inferred GO categories of the second protein match the Golgi apparatus	0.0279	0.0357	0.0433
goloc_score_vac2	MultiLoc2 score for whether inferred GO categories of the second protein match the vacuole (plant and fungus)	0.0399	0.0330	N/A
phyloloc_score_nuc2	MultiLoc2 score for whether phylogenetic profile of second protein BLAST hits indicates nuclear localization	0.0443	0.0435	0.0476
phyloloc_score_cyt2	MultiLoc2 score for whether phylogenetic profile of second protein BLAST hits indicates cytoplasmic localization	0.0414	0.0473	0.0492
phyloloc_score_mit2	MultiLoc2 score for whether phylogenetic profile of second protein BLAST hits indicates mitochondrial localization	0.0500	0.0548	0.0512
phyloloc_score_chl2	MultiLoc2 score for whether phylogenetic profile of second protein BLAST hits indicates chloroplast localization (plant)	0.0451	N/A	N/A
phyloloc_score_ext2	MultiLoc2 score for whether phylogenetic profile of second protein BLAST hits indicates extracellular localization	0.0509	0.0475	0.0483
phyloloc_score_pm2	MultiLoc2 score for whether phylogenetic profile of second protein BLAST hits indicates plasma membrane localization	0.0497	0.0490	0.0521
phyloloc_score_per2	MultiLoc2 score for whether phylogenetic profile of second protein BLAST hits indicates peroxisomal localization	0.0489	0.0524	0.0491
phyloloc_score_er2	MultiLoc2 score for whether phylogenetic profile of second protein BLAST hits indicates ER localization	0.0498	0.0521	0.0528
phyloloc_score_gol2	MultiLoc2 score for whether phylogenetic profile of second protein BLAST hits indicates Golgi apparatus localization	0.0435	0.0474	0.0506
phyloloc_score_vac2	MultiLoc2 score for whether phylogenetic profile of second protein BLAST hits indicates vacuolar localization (plant and fungal)	0.0502	0.0578	N/A
motif_search_er_target2	MultiLoc2 indicator of ER targeting signal for the second protein	0.0008	0.0056	0.0030

motif_search_pm_receptor_domain2	MultiLoc2 indicator of plama membrane targeting signal for the second protein	0.0000	-0.0044	0.0159
motif_search_peroxi_target2	MultiLoc2 indicator of peroxisomal targeting signal for the second protein	0.0083	0.0062	0.0119
motif_search_nuclear_bipartite2	MultiLoc2 indicator of nuclear bitartite protein for the second protein	0.0094	0.0113	0.0103
motif_search_predictNLS2	MultiLoc2 indicator of nuclear localization signal for the second protein	0.0136	0.0111	0.0127
motif_search_nls_mono2	MultiLoc2 indicator of nuclear localization signal for the second protein	0.0203	0.0168	0.0203
motif_search_dna_associated_domain2	MultiLoc2 indicator of dna associated domain for the second protein	0.0160	0.0019	0.0232
phyloloc_score_lys2	MultiLoc2 score for whether phylogenetic profile of second protein BLAST hits indicates lysosome localization (animal)	N/A	N/A	0.0534
goloc_score_lys2	MultiLoc2 score for whether inferred GO categories of the second protein match the lysosome membrane (animal)	N/A	N/A	0.0418
svm_aac_score_lys2	MultiLoc2 score for whether the overall amino acid content of the second protein matches the lysosome (animal)	N/A	N/A	0.0523

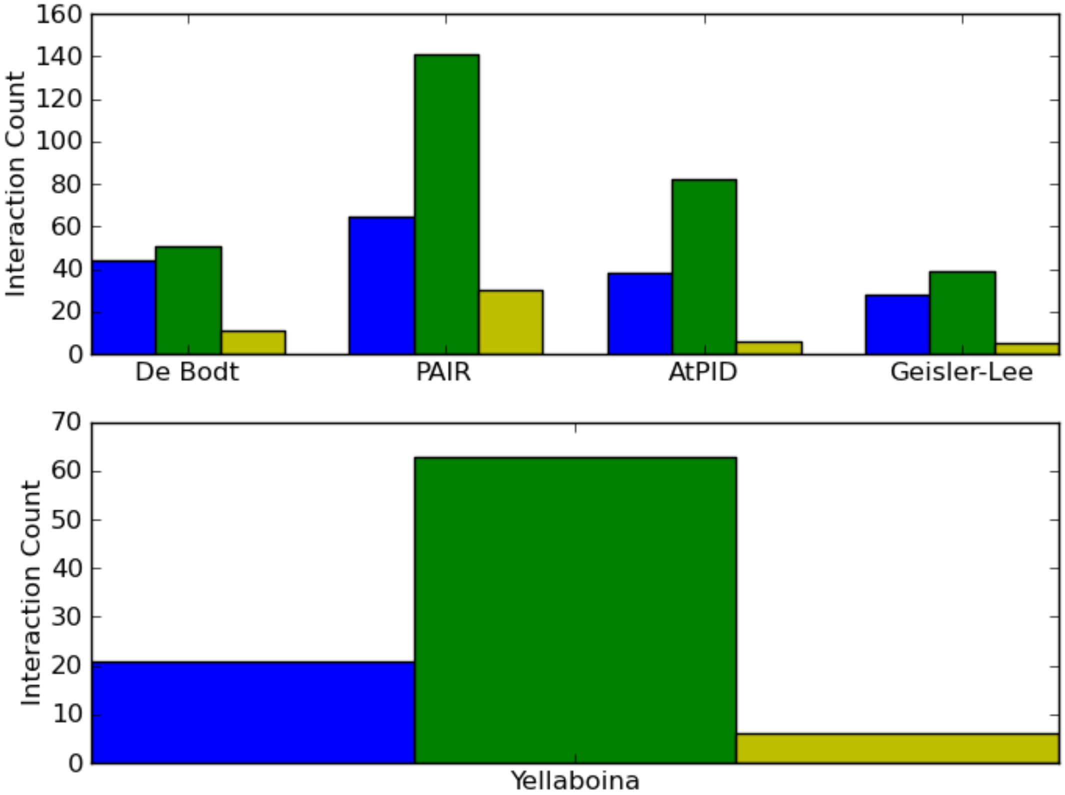

Supplemental Figure 1. The number of positive predictions on sets of novel experimentally-determined interactions for ENTS and several alternative prediction techniques when only allowing predictions involving proteins not contained in any of the training data. This is shown for *A. thaliana* (upper) and *M. musculus* (lower). ENTS results are shown in blue; the alternative classifiers' results are shown in green; and the number of positive predictions shared between each pair of classifiers is shown in yellow. Too few proteins unused for domain-pair odds calculation were available for *S. cerevisiae* and *H. sapiens* to obtain any predicted interactions using the alternative classifiers.

Supplemental Table 2. The total number of predicted interactions, the number of those interactions with experimental support, and the relative frequency of experimentally-supported interactions within this set among novel interactions from a high-throughput all-vs.-all Y2H experiment in *A. thaliana*.

**Comparison**	**Predicted**	**Exp. Positive**	**Ratio**
ENTS/De Bodt	2838/1977	183/87	0.064/0.044
ENTS/PAIR	5839/7355	253/298	0.043/0.041
ENTS/AtPID	1695/839	128/112	0.075/0.133
ENTS/Geisler-Lee	1454/921	114/54	0.078/0.059

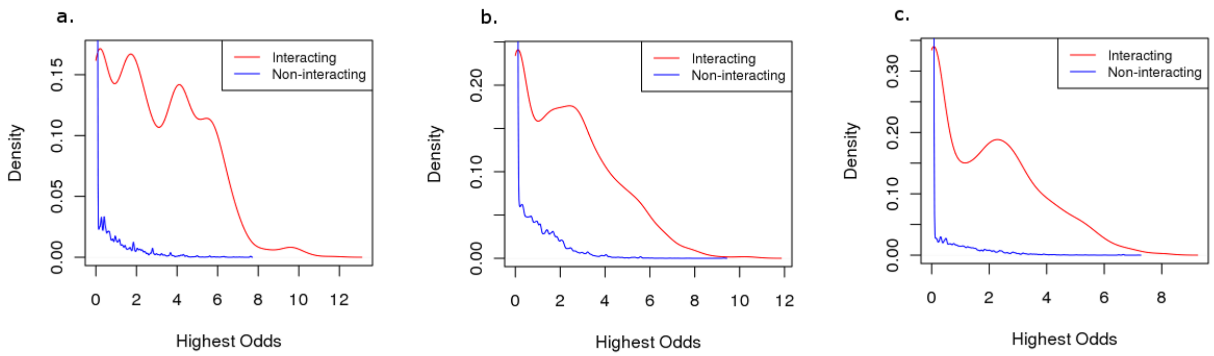

Supplemental Figure 1. Kernel density plots of the highest domain odds scores for interacting and non-interacting protein pairs in *A. thaliana* (a), *H. sapiens* (b), and *S. cerevisiae* (c).

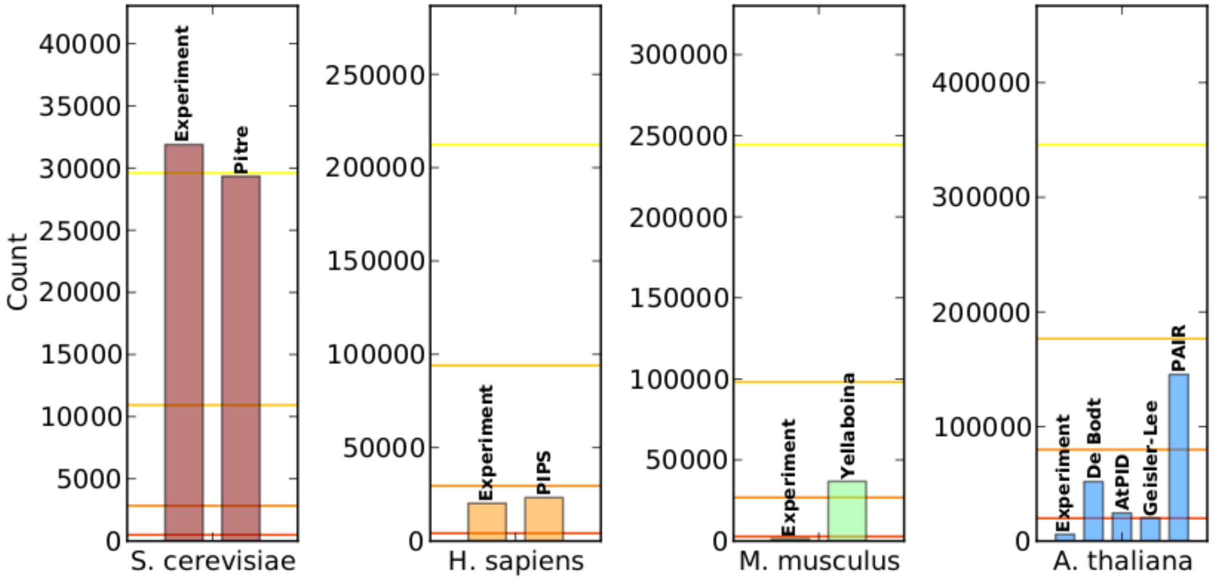

Supplemental Figure 2. The total number of interactions predicted by ENTS compared to other classifiers. ENTS results are shown as horizontal lines, with colors corresponding to confidence thresholds as follows: 0.55 (yellow), 0.65 (light orange, 0.75 (dark orange), 0.85 (red).

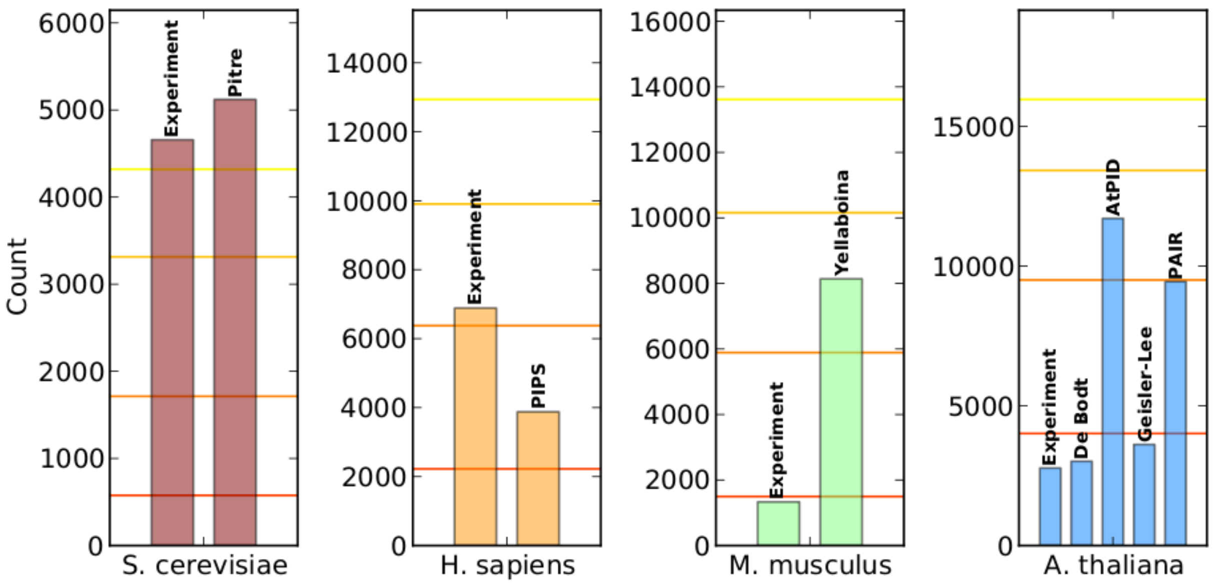

Supplemental Figure 3. The total number of genes with predicted interactions from ENTS compared to other classifiers.

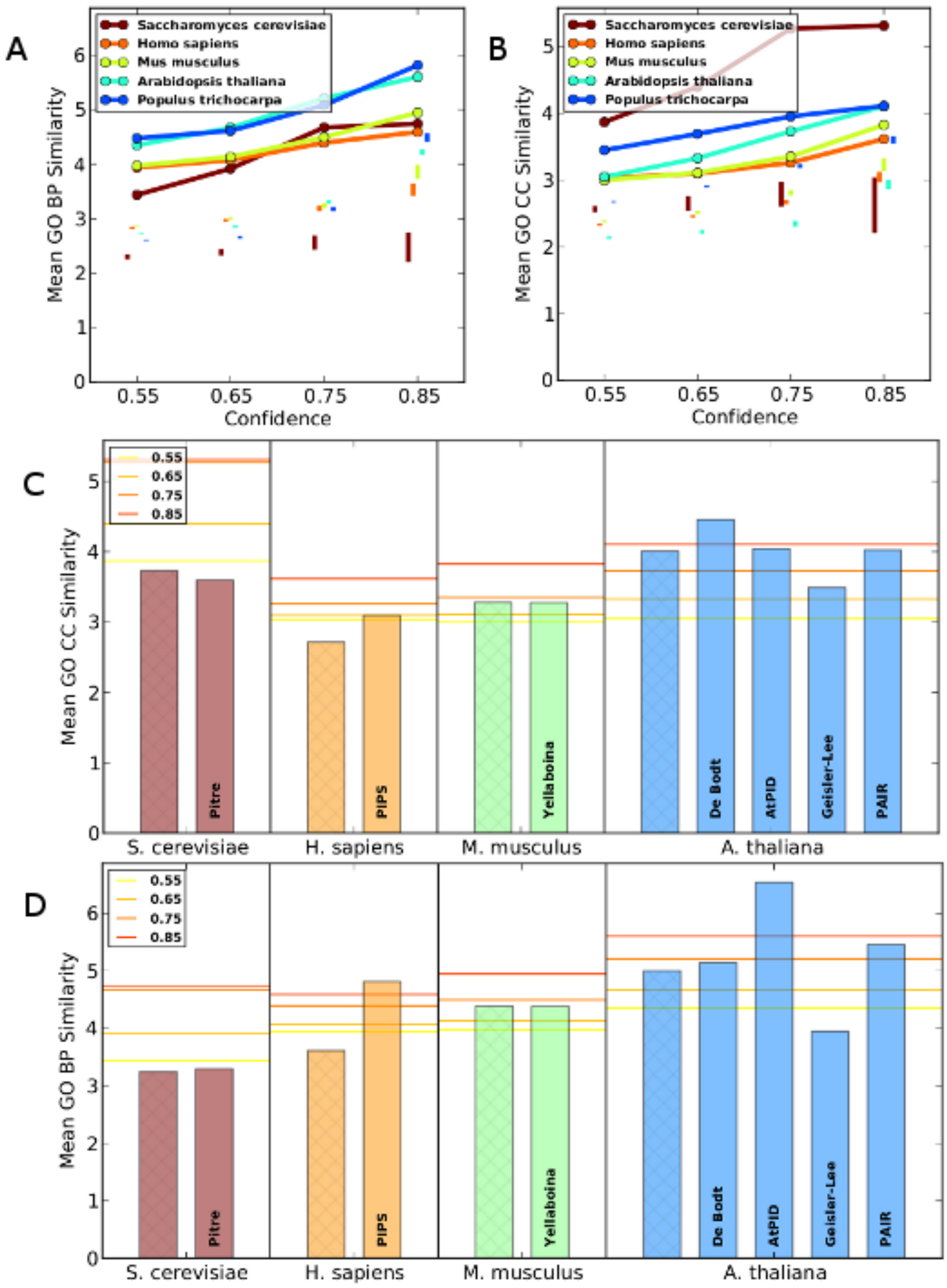

Supplemental Figure 4. (A) The mean GO biological process and (B) cellular component similarity scores for predicted ENTS interactions. Vertical lines at each confidence level show the total range for randomized networks. (D) Shared GO biological process and (C) cellular component similarity for ENTS as compared to other predicted networks.

Supplemental Table 3. 95% confidence intervals for coefficients in the logistic regressions on WGD retention in *P. trichocarpa* and *A. thaliana*. Models were fit using either all genes with at least one interaction in the ENTS predicted network or with the subset with at least 10 neighbors. Confidence intervals were based on non-parametric bootstrapping with 10,000 random resamplings.

**WGD**	**Data**	**Intercept**	**log(degree centrality)**	**duplicated neighbors**	**log(degree centrality):duplicated neighbors**
P. trichocarpa Salicoid	All genes	(-2.46,-1.34)	(-0.31,-0.18)	(4.08,5.77)	(0.48,0.68)
	≥10 neighbors	(-2.41,-0.84)	(-0.31,-0.12)	(3.21,5.58)	(0.38,0.67)
A. thaliana α	All genes	(-2.40,-1.58)	(-0.15,-0.05)	(4.52,6.97)	(0.54,0.85)
	≥10 neighbors	(-3.31,-0.98)	(-0.26,0.10)	(3.68,9.80)	(0.27,1.21)
A. thaliana β/γ	All genes	(-2.87,-2.14)	(-0.09,0.01)	(5.50,8.42)	(0.55,0.90)
	≥10 neighbors	(-4.18,-2.31)	(-0.31,-0.01)	(7.65,18.14)	(0.88,2.55)

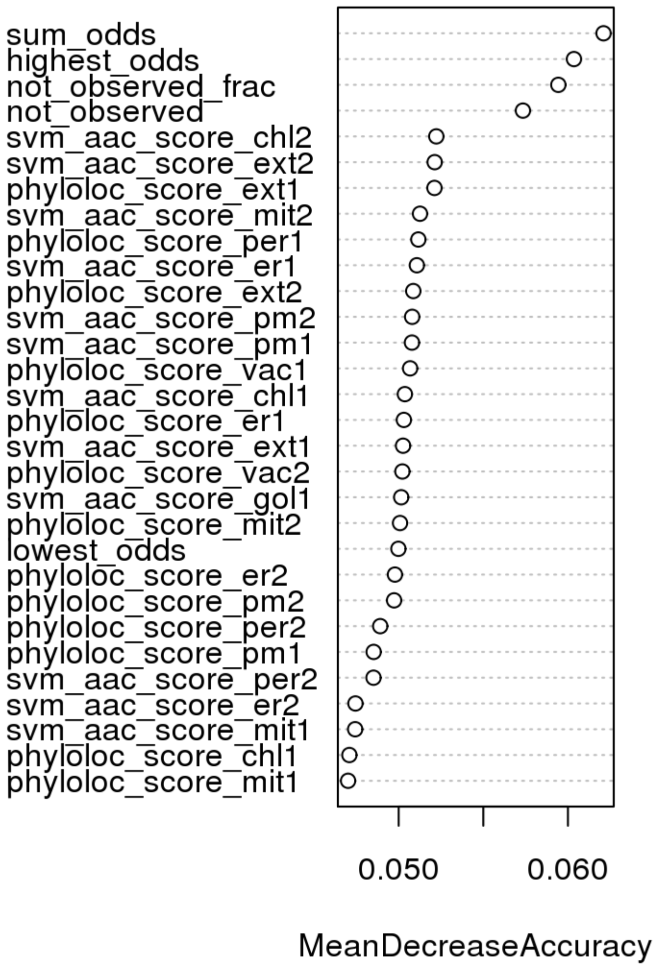

Supplemental Figure 6. Variable importance for the first 30 most important variables in the *A. thaliana*-trained ENTS classifier. Importance was measured as the mean decrease in OOB classification accuracy during training following permutation of the given variable.

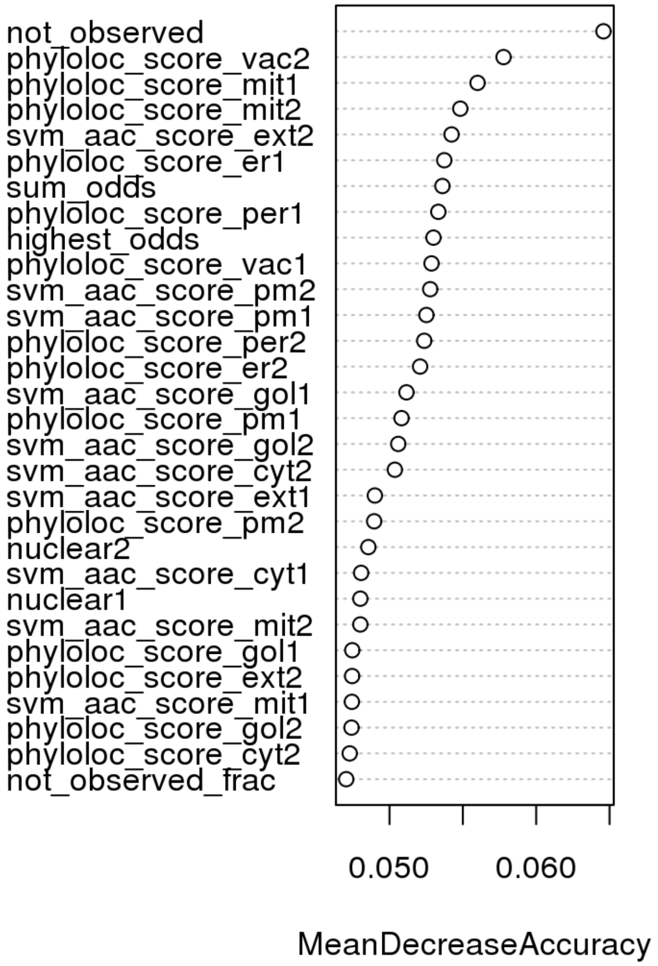

Supplemental Figure 7. Variable importance for the first 30 most important variables in the *S. cerevisiae*-trained ENTS classifier.

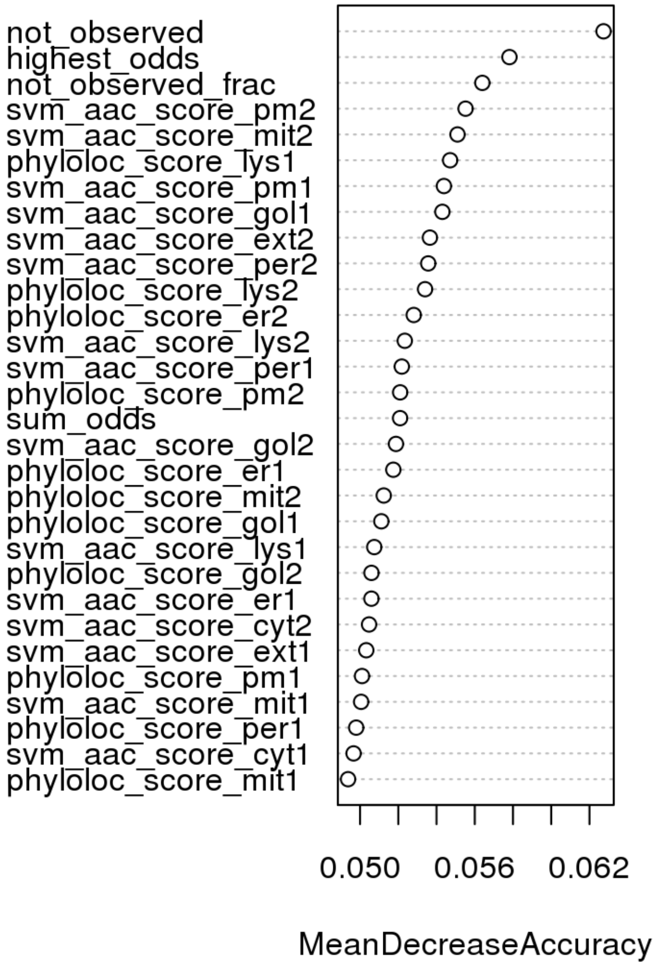

Supplemental Figure 8. Variable importance for the first 30 most important variables in the *H. sapiens*-trained ENTS classifier.
